# Comparative Effectiveness of Intracranial Pressure Monitoring on 6-Month Outcomes of Critically Ill Patients With Traumatic Brain Injury

**DOI:** 10.1001/jamanetworkopen.2023.34214

**Published:** 2023-09-27

**Authors:** Giovanni Nattino, Lorenzo Gamberini, Obou Brissy, Greta Carrara, Randall Chesnut, Valentina Chiarini, Arturo Chieregato, Akos Csomos, Joanne M. Fleming, Primoz Gradisek, Rafael Kaps, Theodoros Kyprianou, Isaac Lazar, Stanley Lemeshow, Malgorzata Mikaszewska-Sokolewicz, Giulia Paci, Carlotta Rossi, Nancy Temkin, Nektaria Xirouchaki, Aimone Giugni, Guido Bertolini

**Affiliations:** 1 Laboratory of Clinical Epidemiology, Department of Public Health, Istituto di Ricerche Farmacologiche Mario Negri IRCCS, Ranica, Bergamo, Italy; 2 Anesthesia, Intensive Care and Prehospital Emergency, Maggiore Hospital, Bologna, Italy; 3 Department of Neurological Surgery and School of Global Health, University of Washington, Seattle; 4 Neurointensive Care Unit, Grande Ospedale Metropolitano Niguarda, Milan, Italy; 5 Hungarian Army Medical Center, Budapest, Hungary; 6 Clinical Department of Anaesthesiology and Intensive Therapy, University Medical Centre Ljubljana, Ljubljana, Slovenia; 7 Faculty of Medicine, University of Ljubljana, Ljubljana, Slovenia; 8 General Hospital Novo Mesto, Novo Mesto, Slovenia; 9 University of Nicosia Medical School, Nicosia, Cyprus; 10 University Hospitals Bristol and Weston NHS Foundation Trust, Bristol, United Kingdom; 11 Pediatric Intensive Care Unit, Soroka Medical Center and Faculty of Health Sciences, Ben-Gurion University of the Negev, Beer Sheva, Israel; 12 Division of Biostatistics, College of Public Health, Ohio State University, Columbus; 13 Clinic of Anaesthesia and Intensive Care, Medical University of Warsaw, Warsaw, Poland; 14 Hospital Nursing Management, AUSL Romagna, Maurizio Bufalini Hospital, Cesena, Italy; 15 Department of Neurological Surgery and Department of Biostatistics, University of Washington, Seattle; 16 University Hospital of Heraklion, Crete, Greece

## Abstract

**Importance:**

While the relationship between persistent elevations in intracranial pressure (ICP) and poorer outcomes is well established for patients with traumatic brain injury (TBI), there is no consensus on how ICP measurements should drive treatment choices, and the effectiveness of ICP monitoring remains unknown.

**Objective:**

To evaluate the effectiveness of ICP monitoring on short- and mid-term outcomes of patients with TBI.

**Design, Setting, and Participants:**

CREACTIVE was a prospective cohort study that started in March 2014 and lasted 5 years. More than 8000 patients with TBI were enrolled at 83 intensive care units (ICUs) from 7 countries who joined the CREACTIVE Consortium. Patients with TBI who met the Brain Trauma Foundation guidelines for ICP monitoring were selected for the current analyses, which were performed from January to November 2022.

**Exposure:**

Patients who underwent ICP monitoring within 2 days of injury (exposure group) were propensity score–matched to patients who were not monitored or who underwent monitoring 2 days after the injury (control group).

**Main Outcome and Measure:**

Functional disability at 6 months as indicated by Glasgow Outcome Scale–Extended (GOS-E) score.

**Results:**

A total of 1448 patients from 43 ICUs in Italy and Hungary were eligible for analysis. Of the patients satisfying the ICP-monitoring guidelines, 503 (34.7%) underwent ICP monitoring (median [IQR] age: 45 years [29-61 years]; 392 males [77.9%], 111 females [22.1%]) and 945 were not monitored (median [IQR] age: 66 years [48-78 years]; 656 males [69.4%], 289 females [30.6%]). After matching to balance the variables, worse 6-month recovery was observed for monitored patients compared with nonmonitored patients (death/vegetative state: 39.2% vs 40.6%; severe disability: 33.2% vs 25.4%; moderate disability: 15.7% vs 14.9%; good recovery: 11.9% vs 19.1%, respectively; *P* = .005). Monitored patients received medical therapies significantly more frequently.

**Conclusions and Relevance:**

In this cohort study, ICP monitoring was associated with poorer recovery and more frequent medical interventions with their relevant adverse effects. Optimizing the value of ICP monitoring for TBI requires further investigation on monitoring indications, clinical interventions, and management protocols.

## Key Points

**Question:**

For patients with traumatic brain injury (TBI) who meet the current guidelines for intracranial pressure (ICP) monitoring, is monitoring associated with improved functional recovery?

**Findings:**

In this cohort study involving 1448 patients from 43 intensive care units, patients who underwent ICP monitoring had a worse Glasgow Outcome Scale–Extended score at 6 months than the matched, nonmonitored control patients and received medical therapies significantly more frequently.

**Meaning:**

These findings raise questions about the efficacy and safety of the use of ICP monitoring in driving therapies for intracranial hypertension.

## Teaser

This cohort study examines data from the large international CREACTIVE Consortium to gauge whether monitoring intracranial pressure among patients with traumatic brain injury is associated with better short- and mid-term outcomes.

## Introduction

Traumatic brain injury (TBI) remains a worldwide public health challenge.^1,2,3^ Elevated intracranial pressure (ICP) is a frequent consequence of severe TBI (sTBI).^4^ The injury triggers primary and secondary pathophysiological processes, possibly leading to uncontrolled intracranial hypertension. Untreated, this condition results in brain structure herniation, brainstem compression, and brain ischemia, each associated with increased mortality and worse functional outcomes.^4,5,6^

Intracranial pressure monitoring has consequently been advocated in sTBI management to detect intracranial hypertension and guide its treatment.^7,8,9,10^ However, while the association between higher ICP and poorer outcomes is generally accepted,^5,11^ determining indications for ICP monitoring and the effectiveness of therapy driven by ICP monitoring remains controversial.^12,13,14,15,16,17,18^ According to the most recent Brain Trauma Foundation guidelines, the efficacy of ICP monitoring on clinical outcomes is supported by low-quality evidence.^19^ Previous studies have provided contradictory results,^12,16,17,18,20,21,22,23,24,25,26,27,28^ including 1 randomized clinical trial (RCT) where care driven by ICP monitoring was not found to be superior to care based on imaging and neurologic examination.^20^

Our study evaluates the comparative effectiveness of ICP monitoring on 6-month functional outcomes as measured by the Glasgow Outcome Scale–Extended (GOS-E),^29^ for patients with TBI who meet Brain Trauma Foundation monitoring criteria. To address this question, we leveraged the database of the CREACTIVE (Collaborative Research on Acute Traumatic Brain Injury in Intensive Care Medicine in Europe) Consortium. CREACTIVE is an international prospective observational study aimed at describing the epidemiology of TBI in Europe and improving the quality of care in the field.^30^

## Methods

### Study Design

We selected eligible patients from the database of the CREACTIVE Consortium, which was joined by 83 intensive care units (ICUs) from 7 countries (Cyprus, Greece, Hungary, Israel, Italy, Poland, and Slovenia). Participating centers prospectively collected data on 8179 patients admitted to the ICU after experiencing TBI between 2014 and 2019,^31^ including demographic data, comorbidities, trauma characteristics, clinical conditions at the scene and on ICU admission, details of the worst computed tomography (CT) scan in the first 48 hours posttrauma, neurosurgical procedures, treatments administered in the ICU, complications, and ICU and hospital mortality. Data quality was ensured by advanced operating procedures (eAppendix 1 in Supplement 1).

The study was approved by the Ethics Committee Lazio 1 (Rome, Italy) and the institutional review boards of participating centers. Informed consent was obtained from patients or their legal representatives. Where national legislation so permitted, a waived or delayed consent process was implemented for patients in a coma or experiencing high-stress levels. The results are presented according to the Strengthening the Reporting of Observational Studies in Epidemiology (STROBE) reporting guideline.^32^

### Inclusion and Exclusion Criteria

We selected adults admitted to ICUs with in-hospital availability of neurosurgery facilities. We excluded patients who were admitted to pediatric ICUs (eliminating all centers from Israel, where only pediatric ICUs joined the consortium). We also excluded patients admitted to ICUs on or after the third day after injury, admissions for palliative sedation or organ donation, and patients with preexisting functional disabilities. We excluded patients arriving at the emergency department with bilaterally dilated, nonreactive pupils, as we assumed that their very high mortality rates would be only marginally influenced by ICP monitoring. We selected only the first admission in case of multiple registrations of the same patient from different ICUs (eg, after transfers).

Within the identified cohort, we selected the patients satisfying criteria from the Brain Trauma Foundation guidelines for ICP monitoring,^19^ ie, patients with sTBI (Glasgow Coma Scale score 3-8), an abnormal CT scan (Marshall CT classification 2 or higher), and/or at least 2 of the following conditions: older than 40 years, abnormal motor response, systolic blood pressure less than 90 mm Hg, or clinically relevant hypotension.

### ICP Monitoring

Data collection included information on whether ICP was monitored and, if so, when monitoring started. The treatment group included all patients whose monitoring began within 2 days of the injury. The control group consisted of all patients who were never monitored or whose monitoring was initiated after the second day.

### Outcomes

The primary outcome was 6-month GOS-E score.^29^ ICU staff, who were blinded to the aim of this study, assessed the scale via telephone follow-up interviews. The staff was trained through a dedicated 2-day course. ICU and hospital mortality were the secondary outcomes.

Patients lost to the 6-month follow-up were excluded from the analyses. To evaluate the effect of this exclusion on the study results, we performed the sensitivity analysis described in eAppendix 2 in Supplement 1.

### Statistical Analysis

Analyses for this substudy were performed from January to November 2022. We used a propensity score–matched design. Propensity score matching is a robust methodology to estimate causal effects in observational studies.^33^ Matching patients via propensity scores establishes treatment and control groups that are well balanced for all factors associated with both the decision to initiate ICP monitoring and patient outcome. To control for any bias possibly introduced by the unbalanced distribution of the study countries, characterized by different patient outcomes and TBI management policies, we only matched patients treated in the same country and excluded countries where high-quality matching was impossible. Patients were thus matched on the propensity score within a country and within the value of 3 variables that were deemed as critical: age group, mass lesion in the CT scan, and prehospital hypotension.

We used the full matching algorithm,^34^ which creates matched sets with a variable number of treated and control patients. This approach allows for the loss of very few (if any) eligible patients from matching, thereby avoiding uncontrolled, unaware selection biases as a result of the matching process. It requires weighted postmatching analyses, where the weights depend on the size and composition of the matched sets.^35^ We evaluated the quality of matching in terms of the weighted standardized mean differences in pretreatment variables and considered differences smaller than 10% as negligible discrepancies. We performed analyses using R version 4.0.2 (R Project for Statistical Computing). *P* values were considered significant if less than .05. Further methodological details are provided in eAppendix 3 in Supplement 1.

## Results

### Patients

**Figure 1.**
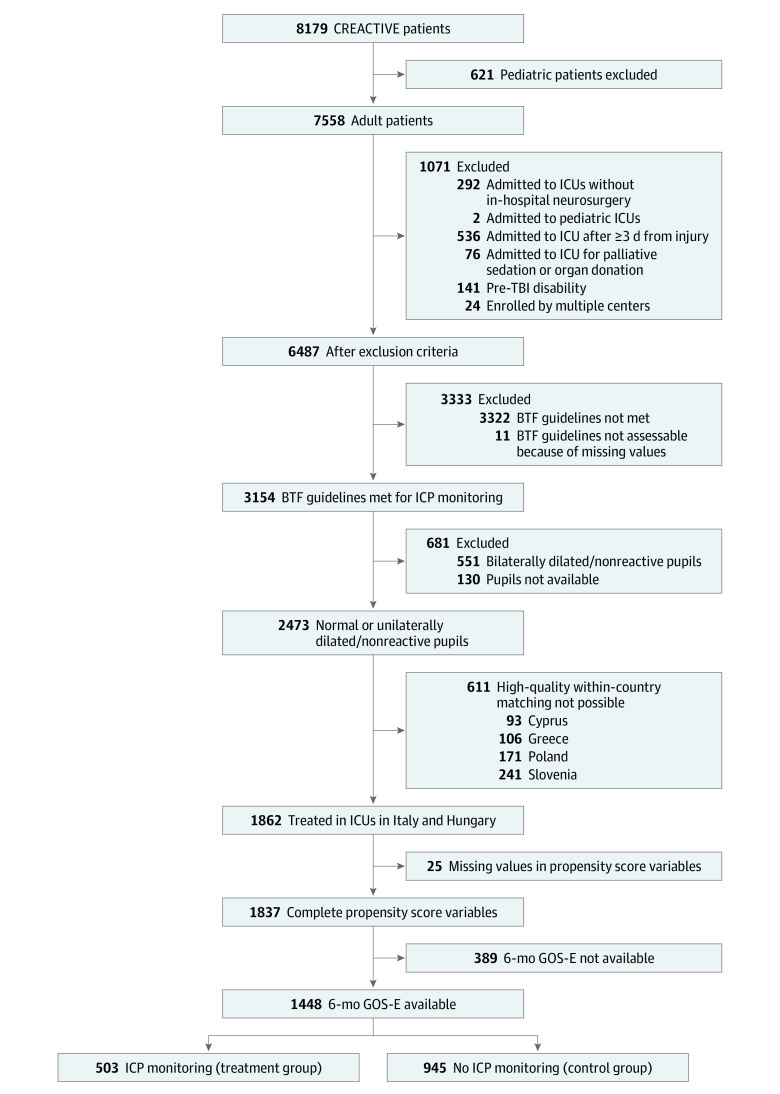
Flowchart Describing the Patient Selection BTF indicates Brain Trauma Foundation; CREACTIVE, Collaborative Research on Acute Traumatic Brain Injury in Intensive Care Medicine in Europe; GOS-E, Glasgow Outcome Scale–Extended score; ICP, intracranial pressure; ICU, intensive care unit; TBI, traumatic brain injury.

Figure 1 describes the sample selection. Of the 6487 patients remaining after exclusion criteria were applied, 3154 (48.7%) met the Brain Trauma Foundation guidelines for ICP monitoring, and 2473 of them (78.4%) did not present with bilaterally dilated pupils on arrival to the emergency department. As expected, hospital mortality in this subgroup of excluded patients was very high (80.1%).

First, we sought to match patients in the cohort by including all the study countries. Table 1 provides the distribution of the patients in the treatment groups for each country after excluding patients lost at 6-month follow-up and with missing values in the propensity score covariates. We observed very different proportions of patients undergoing ICP monitoring across countries, ranging from 3.6% (Poland) to 66.5% (Slovenia). Unfortunately, the analysis involving all countries produced a matched sample with poorly balanced pretreatment covariates, as indicated by the large standardized mean differences (eAppendix 4 in Supplement 1). This poor balance, precluding any meaningful comparison of the outcomes, was attributed to the limited size of the control groups in 3 countries (Cyprus, Greece, and Slovenia), where more than 50% of eligible patients received ICP monitoring (Table 1). Thus, we excluded patients from these countries and those from Poland, where the extremely low proportion of treated patients suggested that the decision to monitor ICP followed different criteria from those applied in the other countries.

**Table 1.** Distribution of Patients Across Countries Involved in the CREACTIVE Consortium

Country	No. (row %)	
	No ICP monitoring	ICP monitoring
Cyprus	24 (36.9)	41 (63.1)
Greece	38 (42.2)	52 (57.8)
Hungary	160 (58.4)	114 (41.6)
Italy	785 (66.9)	389 (33.1)
Poland	135 (96.4)	5 (3.6)
Slovenia	75 (33.5)	149 (66.5)

Abbreviations: CREACTIVE, Collaborative Research on Acute Traumatic Brain Injury in Intensive Care Medicine in Europe; ICP, intracranial pressure.

The analysis was therefore limited to the 1448 patients (73.6%) admitted to 36 ICUs in Italy and 7 ICUs in Hungary. Of them, 503 patients (34.7%) underwent ICP monitoring within the first 2 days of their injury and formed the treatment group (median [IQR] age, 45 years [29-61 years]; 392 males [77.9%], 111 females [22.1%]), while the 12 patients (0.8%) who started the monitoring after the second day and the 933 nonmonitored patients (64.4%) formed the control group (median [IQR] age, 66 years [48-78 years]; 656 males [69.4%], 289 females [30.6%]). Monitored patients were younger, presented fewer comorbidities, had more injuries in body areas other than the head, and underwent surgical interventions more frequently (Table 2). The distribution across ICUs is presented in eAppendix 5 in Supplement 1, which reveals heterogeneous use of the procedure (median percentage of monitored patients: 30.0%; IQR, 21.8%-50.0%).

**Table 2.** Demographic and Clinical Characteristics at ICU Admission of Eligible Patients

Variables	No ICP monitoring		ICP monitoring, No. (%)	*P* value^b^
	All, No. (%)	Weighted distribution, %^a^		
No. of patients	945	503	503	
Age, y				
Mean (SD)	61.9 (20.1)	46.4 (18.3)	45.9 (18.5)	.67
Median (IQR)	66.0 (48.0-78.0)	44.0 (32.0-60.0)	45.0 (29.0-61.0)	
Sex				
Female	289 (30.6)	18.5	111 (22.1)	.11
Male	656 (69.4)	81.5	392 (77.9)	
Comorbidities				
Any comorbidity^c^	601 (63.6)	39.5	176 (35.0)	.13
Antiplatelet therapy	107 (11.3)	5.3	26 (5.2)	.94
COPD	47 (5.0)	2.4	12 (2.4)	.97
Dementia	29 (3.1)	0.4	2 (0.4)	>.99
Drug-induced coagulopathy	61 (6.5)	2.7	14 (2.8)	.90
Heart failure	31 (3.3)	0.6	7 (1.4)	.46
Liver disease	34 (3.6)	3.1	10 (2.0)	.21
Renal disease	32 (3.4)	0.6	3 (0.6)	.99
Penetrating trauma	24 (2.5)	4.1	25 (5.0)	.54
Pretreatment GCS score				
Mean (SD)	5.4 (1.9)	5.1 (1.8)	5.2 (1.8)	.09
Median (IQR)	6.0 (3.0-7.0)	5.0 (3.0-7.0)	5.0 (3.0-7.0)	
Main lesion				
Cerebral contusion/laceration	211 (22.3)	32.7	159 (31.6)	.69
Extradural/epidural hematoma	41 (4.3)	7.3	38 (7.6)	.87
Traumatic subdural hematoma	360 (38.1)	28.2	139 (27.6)	.83
Intraparenchymal bleeding	86 (9.1)	9.0	53 (10.5)	.43
Diffuse injury without edema	101 (10.7)	10.7	40 (8.0)	.07
Diffuse injury with edema	21 (2.2)	5.7	39 (7.8)	.28
Subarachnoid hemorrhage	115 (12.2)	6.0	33 (6.6)	.65
Skull fracture	10 (1.1)	0.5	2 (0.4)	.97
Injuries other than TBI^d^				
Abdomen	95 (10.1)	12.7	61 (12.1)	.80
Chest	269 (28.5)	38.6	213 (42.3)	.24
Pelvis, bones, joints, and muscles	222 (23.5)	25.2	134 (26.6)	.55
Major vessels	25 (2.6)	4.0	19 (3.8)	.87
Spine	184 (19.5)	25.8	142 (28.2)	.38
Other	3 (0.3)	0.3	3 (0.6)	.90
Pupils at ED arrival				
Bilaterally reactive/miotic	630 (66.7)	65.1	334 (66.4)	.65
Unilaterally dilated/nonreactive	315 (33.3)	34.9	169 (33.6)	
Hypotension				
Yes	169 (17.9)	17.7	89 (17.7)	.62
No	724 (76.6)	78.5	390 (77.5)	
Information not available	52 (5.5)	3.8	24 (4.8)	
Hypoxia				
Yes	276 (29.2)	34.2	169 (33.6)	.94
No	599 (63.4)	58.9	301 (59.8)	
Information not available	70 (7.4)	6.9	33 (6.6)	
Transfer from other ICU for hospital expertise	14 (1.5)	2.5	13 (2.6)	.94
Surgery before ICU admission	433 (45.8)	61.4	308 (61.2)	.95
Neurosurgery within 2 d from injury^e^	316 (33.4)	42.4	231 (45.9)	.30
Cardiovascular failure on ICU admission				
None	588 (62.2)	37.5	188 (37.4)	.67
Without shock	152 (16.1)	31.7	171 (34.0)	
With shock	205 (21.7)	30.8	144 (28.6)	
Metabolic failure on ICU admission	214 (22.6)	26.6	127 (25.2)	.62
Kidney failure on ICU admission	142 (15.0)	7.0	39 (7.8)	.62
Worst CT scan of the first 48 h in ICU				
Marshall scale				
Diffuse injury 1	94 (9.9)	4.1	19 (3.8)	.59
Diffuse injury 2	338 (35.8)	35.6	163 (32.4)	
Diffuse injury 3	70 (7.4)	14.0	84 (16.7)	
Diffuse injury 4	42 (4.4)	2.7	18 (3.6)	
Mass lesion (5 or 6)	401 (42.4)	43.5	219 (43.5)	
Midline shift >5 mm	351 (37.1)	33.3	163 (32.4)	.76
Lesion volume >25 mL	335 (35.4)	35.8	181 (36.0)	.96
Petechiae	402 (42.5)	50.9	266 (52.9)	.55
Cistern condition				
Normal	424 (44.9)	44.3	202 (40.2)	.39
Compressed or distorted	361 (38.2)	44.3	243 (48.3)	
Absent	160 (16.9)	11.4	58 (11.5)	

Abbreviations: COPD, chronic obstructive pulmonary disease; CT, computed tomography; ED, emergency department; GCS, Glasgow Coma Scale; ICP, intracranial pressure; ICU, intensive care unit; TBI, traumatic brain injury.

^a^ Data for patients in the no ICP monitoring group are weighted to make them comparable with those in the ICP monitoring group with respect to pretreatment covariates. Weights are defined by the matched design.

^b^ *P* value of the weighted tests comparing the no ICP monitoring and ICP monitoring groups.

^c^ The full list of comorbidities collected in the case report form is provided in eAppendix 7 in Supplement 1.

^d^ The complete list of lesions considered in each body region is reported in eAppendix 8 in Supplement 1.

^e^ For the patients in the ICP monitoring group, number of neurosurgeries performed before or on the same day of the start of the ICP monitoring. For the patients in the no ICP monitoring group, number of neurosurgeries performed before or on the second day of the injury.

### Matching

A total of 31 variables were identified as important matching factors and included in the propensity score model (eAppendix 6 in Supplement 1). Patients were matched according to the estimated propensity score. A total of 247 control patients had a propensity score smaller than the lowest value of the monitored cohort and were not matched. The remaining control patients were assigned a weight defined by the matched structure to render treatment and control groups comparable in pretreatment variables. All the weighted standardized mean differences in the propensity score variables were lower than 10%, suggesting the adequate balance of key covariates (eAppendix 6 in Supplement 1). Table 2 reports the weighted distribution of demographic characteristics, trauma characteristics, and clinical conditions at ICU admission for control patients. Notably, after weighting, control patients closely resembled patients receiving ICP monitoring with respect to all of the considered characteristics. Interestingly, the 2 groups were also similar in terms of the structural characteristics of the admitting hospitals (eAppendix 6 in Supplement 1).

### Outcomes

Table 3 describes the administered interventions, ICU complications, and patient outcomes. After weighting, monitored patients received significantly more medical therapies than nonmonitored patients. The groups were more similar for surgical interventions. Respiratory complications and infections were significantly more common in monitored patients, with other complications being similar.

**Table 3.** Interventions and Patient Outcomes by Treatment Group

Variables	No ICP monitoring		ICP monitoring, No. (%)	*P* value^b^
	All, No. (%)	Weighted distribution, %^a^		
No. of patients	945	503	503	
ICU treatments for intracranial hypertension				
Hypothermia	2 (0.2)	0.1	7 (1.4)	.70
Barbiturate infusion for refractory ICP	9 (1.0)	4.7	62 (12.3)	.02
Hyperventilation PaCO_2_ <25 mm Hg	31 (3.3)	2.8	38 (7.6)	<.001
Indomethacin	1 (0.1)	0.1	12 (2.4)	.67
Mannitol	163 (17.2)	24.2	203 (40.4)	<.001
Hypertonic saline	58 (6.1)	7.7	189 (37.6)	<.001
Sedation/analgesia	355 (37.6)	45.1	343 (68.2)	<.001
Propofol	83 (8.8)	11.4	123 (24.5)	<.001
Subdural hematoma evacuation^c^	171 (20.0)	20.6	91 (20.6)	.99
Extradural hematoma evacuation^c^	23 (2.7)	4.8	44 (10.0)	.009
Lobectomy or contusion removal^c^	13 (1.5)	2.9	30 (6.8)	.03
Primary decompression^d^	88 (9.3)	19.1	94 (18.8)	.89
Secondary decompression^d^	15 (1.6)	4.8	30 (6.0)	.50
Complications during ICU stay				
Cardiovascular	117 (12.4)	10.6	56 (11.1)	.76
Gastrointestinal	21 (2.2)	2.5	17 (3.4)	.37
Neurologic^e^	287 (30.4)	32.8	186 (37.0)	.14
Respiratory	138 (14.6)	19.7	129 (25.6)	.01
Other	37 (3.9)	5.0	33 (6.6)	.27
Infections	276 (29.2)	40.6	299 (59.4)	<.001
ICU outcome				
Dead	311 (32.9)	25.8	125 (24.9)	.72
Conditions at discharge^f^				
Follow simple commands	384 (62.0)	67.8	199 (53.2)	<.001
Cannot follow simple commands	235 (38.0)	32.2	175 (46.8)	
Missing	15		4	
Discharge status^f^				
Ward	221 (34.9)	36.5	94 (24.9)	<.001
Other ICU	206 (32.5)	29.6	98 (25.9)	
High dependency unit	135 (21.3)	16.5	109 (28.8)	
Rehabilitation	72 (11.4)	17.4	77 (20.4)	
Hospital outcome				
Alive	527 (55.9)	67.8	350 (70.0)	.41
Dead	416 (44.1)	32.2	150 (30.0)	
Missing, No.	2		3	
Mechanical ventilation, median (IQR), d				
Alive after ICU	6.0 (2.0-11.5)	8.0 (3.0-16.0)	13.0 (9.0-20.0)	<.001
Deaths in ICU	2.0 (1.0-6.0)	2.0 (1.0-5.0)	5.0 (2.0-10.0)	<.001
Missing, No.	14		2	
ICU stay, median (IQR), d				
Alive after ICU	8.0 (4.0-16.0)	12.0 (5.0-20.0)	18.0 (12.0-26.0)	<.001
Deaths in ICU	3.0 (1.0-6.5)	3.0 (1.0-6.0)	6.0 (2.0-10.0)	<.001
Missing, No.	1		0	
Hospital stay, median (IQR), d				
Alive after ICU	15.0 (8.0-30.0)	20.0 (10.0-34.0)	27.0 (17.8-39.0)	.05
Missing, No.	1		2	
GOS-E status at 6 mo (score)				
Dead (1)	471 (49.8)	35.3	167 (33.2)	.005
Vegetative state (2)	33 (3.5)	5.3	30 (6.0)	
Lower severe disability (3)	145 (15.3)	18.6	125 (24.9)	
Upper severe disability (4)	64 (6.8)	6.9	42 (8.3)	
Lower moderate disability (5)	64 (6.8)	4.6	39 (7.8)	
Upper moderate disability (6)	70 (7.4)	10.4	40 (8.0)	
Lower good recovery (7)	67 (7.1)	7.6	29 (5.8)	
Upper good recovery (8)	61 (6.5)	11.5	31 (6.2)	

Abbreviations: GOS-E, Glasgow Outcome Scale–Extended; ICP, intracranial pressure; ICU, intensive care unit.

^a^ Data for patients in the no ICP monitoring group are weighted to make them comparable with those in the ICP monitoring group with respect to pretreatment covariates. Weights are defined by the matched design.

^b^ *P* value of the weighted tests comparing the no ICP monitoring and ICP monitoring groups.

^c^ The information is missing for 92 patients in the no ICP monitoring group and 62 patients in the ICP monitoring group.

^d^ The information is missing for 2 patients in the ICP monitoring group.

^e^ Neurologic complications include episodes of dilated pupils unreactive to light and brain edema.

^f^ Percentages in these rows were calculated among the number of patients alive.

Comparing monitored with nonmonitored patients after weighting revealed no differences in mortality at ICU discharge (24.9% vs 25.8%, respectively) and hospital discharge (30.0% vs 32.2%, respectively). Significantly fewer monitored patients followed simple commands at ICU discharge (53.2% vs 67.8%, respectively). Length of ICU and hospital stay and duration of mechanical ventilation were longer for monitored patients.

**Figure 2.**
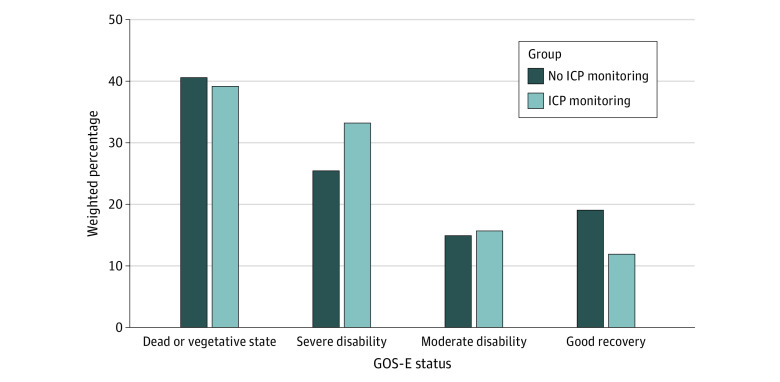
Comparison of the Weighted Distribution of 6-Month Glasgow Outcome Scale–Extended (GOS-E) Score (Grouped in 4 Status Levels) Between the Treatment Groups *P* = .005 for the comparison. ICP indicates intracranial pressure.

Table 3 presents the weighted distribution of the 6-month GOS-E score in the 2 groups, while Figure 2 compares a 4-class grouping of the 8 GOS-E levels. Although mortality rates were similar, we observed worse functional outcomes for the monitored group, with a higher proportion of severe disabilities and a lower proportion of good recoveries (death/vegetative state: 39.2% vs 40.6%; severe disability: 33.2% vs 25.4%; moderate disability: 15.7% vs 14.9%; good recovery: 11.9% vs 19.1%, respectively; *P* = .005). Similar results emerged from our sensitivity analysis to assess the effect of excluding patients with a missing 6-month GOS-E score (eAppendix 2 in Supplement 1).

## Discussion

Although contradictory literature on the efficacy of ICP monitoring in sTBI provides the ideal setting for a large-scale RCT, performing such a study in high-income countries appears unworkable because ICP monitoring is widely perceived as an essential component of sTBI management.^19,36^ Additionally, ICP monitoring is not amenable to direct evaluation and can only be tested as part of a comprehensive protocol that includes the therapeutic options used in response to monitored values. Simultaneously evaluating multiple ICP-monitor–based protocols against a nonmonitored control would require a huge, highly complex RCT protocol, which might still not fully answer the question.

A productive way to explore the issue of ICP monitoring is to interrogate multicenter, prospective, observational studies explicitly conceived for this purpose, such as CREACTIVE. This approach has 3 important advantages. First, it allows for assessing the procedure’s effectiveness in current clinical practice rather than its efficacy in highly controlled environments. Second, collecting data on the use of the numerous treatments for intracranial hypertension helps us understand how ICP monitoring modifies TBI care. Finally, we can study the epidemiology of the use of ICP monitoring. Such insights are critical to formulating clinically relevant research questions to direct future studies.

In this prospective, observational study conducted at 43 ICUs, only one-third of the patients meeting the Brain Trauma Foundation criteria were actually monitored, and the use of monitoring varied considerably across centers. These results reflect a high degree of uncertainty within the TBI-management community about the procedure. Although the mortality among monitored and nonmonitored patients was similar, the monitored group had significantly more patients with severe disability and fewer with good recovery at 6 months. Monitored patients also received significantly more medical interventions and surgery for epidural hematomas or intraparenchymal mass lesions (eg, contusions). Single-event surgical procedures likely mirror the use of monitoring to determine surgical indications for initially marginal lesions. In contrast, medical interventions reflect a complex interaction among ICP thresholds, choice of treatments, perceived and real underlying TBI pathophysiology, management protocols, and responses to such treatments. We observed a much higher therapeutic intensity level, longer ICU stays, and more respiratory and infectious complications in the monitored group.

Occam’s razor suggests first considering that all our findings are interrelated. Monitoring appears strongly associated with an increase in therapies, with ICP-lowering but also adverse effects. While longer ICU stays and increased therapeutic intensity levels can reasonably explain the higher frequency of respiratory and infectious complications, it is unclear why they would increase morbidity without altering mortality. Because the concept comes from a large, multicenter, well-matched study, the issue of treatment toxic effects, possibly in patient subgroups, warrants further investigation.

Our findings differ from those of the only RCT comparing ICP-monitor–based to nonmonitor-based sTBI management, the BEST-TRIP trial,^20^ where no significant 6-month outcome differences were found for the primary 21-factor composite outcome measure or the GOS-E score. This discrepancy may be explained by the RCT design of the BEST-TRIP trial, where the patient selection process was controlled and treatments in both groups were protocolized. Such measures were aimed at decreasing treatment variability but also directly influenced the case mix (eg, BEST-TRIP median age was 15 years lower than in our study) and the number and duration of the delivered treatments. Indeed, in the BEST-TRIP trial, the nonmonitored group presented more and longer brain-specific treatments, while we found significantly more treatments in the monitored group.

Besides the BEST-TRIP trial, several observational studies have evaluated the effectiveness of ICP monitoring. Unfortunately, their results were inconclusive because of important methodological limitations and heterogeneous estimates of association. We systematically reviewed the literature, searching for recent studies (published in or after 2012) evaluating the association of ICP monitoring on mortality or functional recovery in TBI. Studies with limited sample size (<1000 participants) were excluded, leading to the selection of 12 studies.^12,16,17,18,21,22,23,24,25,26,27,28^ Most were monocentric^21,23,26^ or applied suboptimal statistical analyses to assess causal effects in observational designs, such as multiple regression adjustment.^12,16,18,22,24^ Four recent studies relied on propensity score matching.^17,23,27,28^ However, while we applied a full matching design to retain all ICP-monitored patients in the analyses, these studies excluded the monitored patients who remained unmatched after the 1:1 matching process. Because such exclusions are based on the uninterpretable propensity score estimates, they result in selections of the target intervention group that are difficult to interpret, precluding the generalizability of the conclusions to the population of all the patients who had their ICP monitored in clinical practice. Moreover, these studies showed estimates of association in opposite directions.^17,23,27,28^ The SYNAPSE-ICU study was another large, observational study that used propensity score inverse probability weighting to estimate the association of ICP monitoring and 6-month GOS-E score.^25^ One limitation of the study was the small set of variables included in the propensity score and balanced in the statistical analyses: ie, age, sex, Glasgow Coma Scale score, primary diagnosis (TBI, subarachnoid hemorrhage, or intracerebral hemorrhage), highly pathologic CT scan, history of cardiovascular or neurologic comorbidities, and country income level (low/middle vs high). This set is certainly not exhaustive of all the prognostic factors affecting the decision to start ICP monitoring, which is what is recommended in propensity score analyses. We leveraged the extensive CREACTIVE data collection to include a larger set of established prognostic factors in the propensity score model and verified their balance in the matched cohort.

Importantly, clinical studies on ICP monitoring reflect only the context in which ICP data are used and do not question the value of knowing ICP values. Our results, as those of the BEST-TRIP trial, are best interpreted as suggesting reconsideration of the clinical use of ICP data.^20,36^ In this context, several issues remain unresolved, such as patient selection for monitoring, appropriateness of universal vs pathophysiology-specific ICP thresholds,^37,38^ algorithmic vs pathophysiology-specific interventions for intracranial hypertension, acute management of ICP elevations (crisis approach) vs an attempt to maintain ICP within an acceptable range (tranquility approach),^39^ and the role of ICP as a stand-alone trigger vs part of a multimodality-based approach. Future investigations of other large observational databases, such as that from the CENTER-TBI Consortium, should aim at validating our findings and addressing these unresolved research questions.

### Limitations

The main limitation of our study is related to its observational nature. While propensity score matching is a well-established method to evaluate causal relationships in observational studies, it relies on the assumption that all confounders are measured and included in the analysis. Our results could be biased if physicians selected more severe patients for monitoring based on uncollected patient characteristics. This issue is universal in nonrandomized investigations. Even though the existence of unobserved confounders cannot be ruled out, our study was designed to minimize the risk of unobserved confounding. Indeed, the data collection was specifically conceived to address this research question so that all known relevant prognostic variables were collected and balanced in the matched groups.

Using data from only 2 of the countries involved in CREACTIVE is another limitation. We controlled for the substantial between-country difference in patient outcomes by matching patients within the country. This strict requirement forced us to exclude 4 countries because of the limited size of the enrolled cohorts and the lack of overlap of monitored and nonmonitored patients. Furthermore, of the included ICUs, only 7 were Hungarian (19.4%). While this selection possibly limits the generalizability of our results, our evidence relies on the data of 43 ICUs and is robust to the potential bias that could have been introduced if we had matched patients from different countries.

About 20% of the patients were lost to follow-up by the 6-month outcome assessment. While this proportion is nontrivial, it is compatible with the one observed in similar recent studies,^40^ and the robustness of our results to the outcome missingness was verified with a sensitivity analysis. This sensitivity analysis relies on the assumption that outcome values were missing at random; ie, the missingness only depended on fully observed variables. The validity of such assumption is supported by the richness of the data set in terms of TBI prognostic factors and their high degree of completeness, making unlikely the existence of unmeasured prognostic variables the source of outcome missingness.

## Conclusions

This study found a significant association between ICP monitoring and worse patient outcomes, which could be explained by the increased use of medical therapies, with their significant adverse effects, among monitored patients. This result does not question the value of knowing the ICP values but how they should be used to improve patient outcome.

## Notes

Supplement 1. **eAppendix 1.** Homogeneity and Quality of the Data **eAppendix 2.** Sensitivity Analysis for Nonmissing Outcome Selection **eAppendix 3.** Extended Methods **eAppendix 4.** Results of the Attempt of Matched Analysis on all Countries **eAppendix 5.** ICP Monitoring Across ICUs **eAppendix 6.** Results of the Matched Analysis on Italy and Hungary **eAppendix 7.** Comorbidities **eAppendix 8.** Injuries

Supplement 2.
Nonauthor Collaborators

Supplement 3.
Data Sharing Statement
