# Supplementary material for: Comparative Effectiveness of Intracranial Pressure Monitoring on 6-Month Outcomes of Critically Ill Patients With Traumatic Brain Injury
Source: JAMA Netw Open. 2023 Sep 27;6(9):e2334214. doi: 10.1001/jamanetworkopen.2023.34214 (PMC10534270; doi:10.1001/jamanetworkopen.2023.34214)
Supplement: Supplement 1. — eAppendix 1. Homogeneity and Quality of the Data eAppendix 2. Sensitivity Analysis for Nonmissing Outcome Selection eAppendix 3. Extended Methods eAppendix 4. Results of the Attempt of Matched Analysis on all Countries eAppendix 5. ICP Monitoring Across ICUs eAppendix 6. Results of the Matched Analysis on Italy and Hungary eAppendix 7. Comorbidities eAppendix 8. Injuries [file jamanetwopen-e2334214-s001.pdf]

## Supplemental Online Content

Nattino G, Gamberini L, Brissy O, et al; CREACTIVE Consortium. Comparative effectiveness of intracranial pressure monitoring on 6-month outcomes of critically ill patients with traumatic brain injury. *JAMA Netw Open*. 2023;6(9):e2334214. doi:10.1001/jamanetworkopen.2023.34214

**eAppendix 1.** Homogeneity and Quality of the Data
**eAppendix 2.** Sensitivity Analysis for Non-missing Outcome Selection
**eAppendix 3.** Extended Methods
**eAppendix 4.** Results of the Attempt of Matched Analysis on all Countries
**eAppendix 5.** ICP Monitoring Across ICUs
**eAppendix 6.** Results of the Matched Analysis on Italy and Hungary
**eAppendix 7.** Comorbidities
**eAppendix 8.** Injuries

This supplemental material has been provided by the authors to give readers additional information about their work.

© 2023 Nattino G et al. *JAMA Network Open.*

**<u>eAppendix 1. HOMOGENEITY AND QUALITY OF THE DATA</u>**

A senior intensivist (see Appendix) is responsible for protocol and data integrity at each ICU. A detailed definition of all items to collect is immediately visible in the layout of the electronic case report form (eCRF) and a comprehensive, user-friendly, fully indexed, online, operative manual can be easily accessed during data entry.

A complex, multidimensional, validation system ensures maximum data quality. A first level of controls is implemented behind the data collection and follows three different rules: grouping, enabling or disabling, and mutually excluding items. Second-level controls come into operation during data collection and include: completeness checks, warnings on borderline values, and errors. They are of five types: validity (e.g., incorrect date); plausibility (e.g., very high body mass index); logical congruency (e.g., hospital discharge cannot precede ICU discharge); clinical congruency (e.g., a patient with acute respiratory distress syndrome - ARDS - cannot have $PaO_2/FiO_2>200$); score congruency (e.g., a patient with brain coma cannot have Glasgow Coma Scale - GCS - >8). The system allows inconsistent or implausible data to be saved but marks the record as problematic.

The data of each individual unit are synchronized with the central server every 12 hours and centrally processed, searching for inconsistencies that cannot be automatically picked up by the system during data input (e.g., the average mortality of patients with a GCS of 3 should not be lower than in patients with a GCS of 4-5). A data quality report with any remaining unsolved queries is produced twice a year and sent to the centers.

To ensure complete patient recruitment, queries are sent to centers with significant heterogeneity in the number of monthly admissions, assessed using the Chi-square test. Finally, to avoid selection bias, patients admitted in months with over 10% incomplete or inconsistent records (presence of errors or unsolved warnings) were excluded.

Site visits are performed by certified monitors on a random sample of participating ICUs to assess the correspondence between medical records and data entered in the eCRF.

After passing the validation system, data from ICUs with at least four months' valid data were merged to form the aggregate database, ready for statistical analyses.

© 2023 Nattino G et al. *JAMA Network Open.*

**eAppendix 2. SENSITIVITY ANALYSIS FOR NON-MISSING OUTCOME SELECTION**

Of the 1,837 patients eligible for the main analysis, we excluded the 389 (21.2%) patients lost at the 6-month follow-up evaluation (see Figure 1 of the paper). In this sensitivity analysis, we aimed to evaluate whether the selection of patients with non-missing outcome impacted the study results.

First of all, we report in Table S.1 the distribution of the pre-treatment covariates for patients with missing and non-missing outcomes. We observed significant differences in key confounders, such as age, whether the patient underwent surgical intervention, primary head injury, cardiovascular failure at ICU admission, and Marshall CT classification. Such differences suggest that the excluded patients were not a random sample of the patients eligible for the analysis.

To enhance the generalizability of our study results, we addressed the missing data problem with a weighting method (Little and Rubin, Statistical Analysis with Missing Data, 2002). First, the probability of a non-missing outcome was estimated, based on the sample including both patients with non-missing and missing outcomes. Second, when performing statistical analyses on the patients with non-missing outcomes, individuals were weighted by the inverse of the estimated probability, reconstructing the result of the analyses on the sample that included all the patients. Since our main analyses, based on a full matching design, already required weighted procedures, we were able to naturally integrate this missing data approach into the main analyses, by considering weights that were the product of the matching weights (making control and treated patients similar) and missing outcome weights (generalizing the estimates to the whole study sample).

We estimated the probabilities of non-missing outcomes using logistic regression. The estimated odds ratios and the corresponding 95% confidence intervals are provided in Table S.2. Model calibration was evaluated with the Hosmer-Lemeshow goodness-of-fit test (statistic: 12.02, degrees of freedom: 8, p-value: 0.15) and the calibration belt (Figure S.1). No evidence of lack of fit emerged from either of the two approaches.

The model was used to compute the estimated probabilities for all 1,448 patients with non-missing outcomes, included in the main analysis. The distribution of these probabilities is reported in the left panel of Figure S.2, which shows that most are in the 80-90% range, with no patients characterized by very small probabilities, which would result in very large weights. The right panel of Figure S.2 depicts the distribution of the weights, defined as the inverse of these probabilities. The weights were standardized so that the total would correspond to the actual sample size.

Before using the product of the matching weights and missing outcome weights to estimate the effect of ICP monitoring on all the eligible patients, regardless of the missingness of the outcome, we verified that the product of the weights balanced all the confounders, using weighted standardized mean differences (Table S.3). All of the values were smaller than 10%, suggesting that the computed weights were sufficient to make the treated and control groups comparable with respect to key confounders. The balance was also evaluated by comparing the distribution of demographic and clinical characteristics on ICU admission after weighting (Table S.4).

Table S.5 compares the primary and secondary outcomes of the study between patients receiving and not receiving ICP monitoring, after weighting for matching and missing outcome weights. These results adjust the estimates for nonrandom treatment allocation and for the selection of patients with non-missing outcomes. Notably, the similarity of these estimates with those presented in the main analysis (Table 3 of the paper), based on complete patients, supports the robustness of our results to the exclusion of patients with non-missing outcomes.

© 2023 Nattino G et al. *JAMA Network Open.*

**Table S.1.** Distribution of demographic and clinical characteristics of the eligible patients at ICU admission, separately for patients with missing and non-missing 6-month outcome.

| Variables | | Missing outcome | Non-missing outcome | p-value |
|---|---|---|---|---|
| N | | 389 | 1448 | |
| Age | | | | < 0.001 |
| | Mean (SD) | 50.5 (20.3) | 56.3 (21.0) | |
| | Median (Q1-Q3) | 50.0 (33.0, 66.0) | 59.0 (40.0, 75.0) | |
| Sex (Female) – N (%) | | 102 (26.2%) | 400 (27.6%) | 0.58 |
| Comorbidities – N (%) | | | | |
| Any comorbidity[3] | | 176 (45.2%) | 777 (53.7%) | 0.003 |
| Antiplatelet therapy | | 28 (7.2%) | 133 (9.2%) | 0.22 |
| COPD | | 13 (3.3%) | 59 (4.1%) | 0.51 |
| Dementia | | 7 (1.8%) | 31 (2.1%) | 0.67 |
| Drug-induced coagulopathy | | 13 (3.3%) | 75 (5.2%) | 0.13 |
| Heart failure | | 8 (2.1%) | 38 (2.6%) | 0.53 |
| Liver disease | | 16 (4.1%) | 44 (3.0%) | 0.29 |
| Renal disease | | 4 (1.0%) | 35 (2.4%) | 0.09 |
| Penetrating trauma – N (%) | | 7 (1.8%) | 49 (3.4%) | 0.11 |
| Pre-treatment GCS – N (%) | | | | 0.99 |
| | Mean (SD) | 5.3 (1.8) | 5.3 (1.9) | |
| | Median (Q1-Q3) | 6.0 (3.0, 7.0) | 6.0 (3.0, 7.0) | |
| Main lesion – N (%) | | | | |
| | Cerebral contusion/laceration | 111 (28.5%) | 370 (25.6%) | 0.23 |
| | Extradural/epidural hematoma | 33 (8.5%) | 79 (5.5%) | 0.03 |
| | Traumatic subdural hematoma | 115 (29.6%) | 499 (34.5%) | 0.07 |
| | Intraparenchymal bleeding | 21 (5.4%) | 139 (9.6%) | 0.009 |
| | Diffuse injury without edema | 45 (11.6%) | 141 (9.7%) | 0.29 |
| | Diffuse injury with edema | 13 (3.3%) | 60 (4.1%) | 0.47 |
| | Subarachnoid hemorrhage | 42 (10.8%) | 148 (10.2%) | 0.74 |
| | Skull fracture | 9 (2.3%) | 12 (0.8%) | 0.01 |
| Injuries other than TBI[4] – N (%) | | | | |
| | Abdomen | 44 (11.3%) | 156 (10.8%) | 0.76 |
| | Chest | 144 (37.0%) | 482 (33.3%) | 0.17 |
| | Pelvis, bones, joints and muscles | 96 (24.7%) | 356 (24.6%) | 0.97 |
| | Major vessels | 9 (2.3%) | 44 (3.0%) | 0.45 |
| | Spine | 85 (21.9%) | 326 (22.5%) | 0.78 |
| | Other | 3 (0.8%) | 6 (0.4%) | 0.37 |
| Pupils at ED arrival – N (%) | | | | 0.009 |
| | Bilaterally reactive/miotic | 286 (73.5%) | 964 (66.6%) | |
| | Unilaterally dilated/non-reactive | 103 (26.5%) | 484 (33.4%) | |
| Hypotension – N (%) | | | | 0.13 |
| Yes | | 61 (15.7%) | 258 (17.8%) | |
| No | | 298 (76.6%) | 1114 (76.9%) | |
| Information not available | | 30 (7.7%) | 76 (5.2%) | |

© 2023 Nattino G et al. *JAMA Network Open.*

| Variables | Missing outcome | Non-missing outcome | p-value |
|---|---|---|---|
| Hypoxia – N (%) | | | 0.17 |
| Yes | 123 (31.6%) | 445 (30.7%) | |
| No | 228 (58.6%) | 900 (62.2%) | |
| Information not available | 38 (9.8%) | 103 (7.1%) | |
| Transfer from another ICU due to hospital expertise – N (%) | 13 (3.3%) | 27 (1.9%) | 0.08 |
| Surgery before ICU admission – N (%) | 169 (43.4%) | 741 (51.2%) | 0.007 |
| Neurosurgery within 2 days from injury[5] – N (%) | 124 (31.9%) | 547 (37.8%) | 0.03 |
| Cardiovascular failure at ICU admission – N (%) | | | < 0.001 |
| None | 252 (64.8%) | 776 (53.6%) | |
| Without shock | 58 (14.9%) | 323 (22.3%) | |
| With shock | 79 (20.3%) | 349 (24.1%) | |
| Metabolic failure at ICU admission – N (%) | 69 (17.7%) | 341 (23.5%) | 0.01 |
| Renal failure at ICU admission – N (%) | 36 (9.3%) | 181 (12.5%) | 0.08 |
| Worst CT scan of the first 48h in ICU – N (%) | | | |
| Marshall scale | | | < 0.001 |
| Diffuse Injury I | 34 (8.7%) | 113 (7.8%) | |
| Diffuse Injury II | 187 (48.1%) | 501 (34.6%) | |
| Diffuse Injury III | 33 (8.5%) | 154 (10.6%) | |
| Diffuse Injury IV | 13 (3.3%) | 60 (4.1%) | |
| Mass lesion (V or VI) | 122 (31.4%) | 620 (42.8%) | |
| Midline shift >5mm | 100 (25.7%) | 514 (35.5%) | < 0.001 |
| Lesion volume >25ml | 87 (22.4%) | 516 (35.6%) | < 0.001 |
| Petechiae | 166 (42.7%) | 668 (46.1%) | 0.22 |
| Cistern condition | | | < 0.001 |
| Normal | 227 (58.4%) | 626 (43.2%) | |
| Compressed or distorted | 124 (31.9%) | 604 (41.7%) | |
| Absent | 38 (9.8%) | 218 (15.1%) | |

© 2023 Nattino G et al. *JAMA Network Open.*

**Table S.2.** Estimated odds ratios (ORs) and corresponding 95% confidence intervals of the logistic regression model estimating the probability of non-missing outcome.

| Variables | | OR (95% CI) |
|---|---|---|
| Age | | 1.15 (1.07,1.23) |
| Sex (female vs. male) | | 0.97 (0.74,1.27) |
| Comorbidities | | |
| | Antiplatelet therapy (Yes vs. No) | 0.96 (0.60,1.53) |
| | COPD (Yes vs. No) | 0.99 (0.51,1.90) |
| | Dementia (Yes vs. No) | 0.76 (0.32,1.84) |
| | Drug-induced coagulopathy (Yes vs. No) | 0.97 (0.51,1.83) |
| | Cardiac disease (Yes vs. No) | 0.73 (0.32,1.69) |
| | Liver disease (Yes vs. No) | 0.67 (0.36,1.25) |
| | Renal disease (Yes vs. No) | 1.91 (0.63,5.81) |
| Penetrating trauma (Yes vs. No) | | 2.00 (0.84,4.78) |
| Pre-treatment GCS | | |
| | 3 | 0.83 (0.56,1.22) |
| | 4-5 | 0.74 (0.49,1.09) |
| | 6-7 | 0.85 (0.59,1.21) |
| | 8 | 1 (ref.) |
| Main lesion | | |
| | Cerebral contusion/laceration | 3.95 (1.45,10.78) |
| | Extradural/epidural hematoma | 2.20 (0.74,6.54) |
| | Traumatic subdural hematoma | 3.49 (1.26,9.70) |
| | Intraparenchymal bleeding | 5.87 (1.98,17.43) |
| | Diffuse injury without edema | 4.89 (1.73,13.84) |
| | Diffuse injury with edema | 4.91 (1.53,15.74) |
| | Subarachnoid hemorrhage | 3.03 (1.14,8.07) |
| | Skull fracture | 1 (ref.) |
| Injuries other than TBI | | |
| | Abdomen (Yes vs. No) | 1.01 (0.67,1.52) |
| | Chest (Yes vs. No) | 0.84 (0.63,1.12) |
| | Pelvis, bones, joints and muscles (Yes vs. No) | 1.05 (0.78,1.43) |
| | Major vessels (Yes vs. No) | 1.43 (0.67,3.05) |
| | Spine (Yes vs. No) | 1.11 (0.81,1.52) |
| Pupils at ED arrival unilaterally dilated/non-reactive (Yes vs. No) | | 1.13 (0.85,1.50) |
| Hypotension (Yes vs. No) | | 1.04 (0.73,1.49) |
| Hypoxia (Yes vs. No) | | 0.94 (0.72,1.22) |
| Transfer from another ICU due to hospital expertise (Yes vs. No) | | 0.52 (0.26,1.06) |
| Surgery before ICU admission (Yes vs. No) | | 1.38 (0.93,2.04) |
| Neurosurgery within 2 days from injury (Yes vs. No) | | 0.79 (0.50,1.27) |
| Cardiovascular failure on ICU admission | | |
| | None | 1 (ref.) |
| | Without shock | 1.67 (1.20,2.32) |
| | With shock | 1.09 (0.77,1.53) |
| Metabolic failure at ICU admission (Yes vs. No) | | 1.36 (0.98,1.87) |

© 2023 Nattino G et al. *JAMA Network Open.*

| Variables | | OR (95% CI) |
|---|---|---|
| Renal failure at ICU admission (Yes vs. No) | | 0.97 (0.63,1.47) |
| Marshall CT classification | | |
| | Diffuse Injury I | 1 (ref.) |
| | Diffuse Injury II | 1.23 (0.61,2.47) |
| | Diffuse Injury III | 1.44 (0.65,3.16) |
| | Diffuse Injury IV | 1.78 (0.65,4.91) |
| | Mass lesion (V or VI) | 1.24 (0.54,2.86) |
| Midline shift >5mm (Yes vs. No) | | 0.83 (0.49,1.42) |
| Lesion volume >25ml (Yes vs. No) | | 1.74 (1.07,2.82) |
| Petechiae (Yes vs. No) | | 1.33 (1.01,1.75) |
| Cistern condition | | |
| | Normal | 1 (ref.) |
| | Compressed or distorted | 1.36 (0.82,2.25) |
| | Absent | 2.67 (1.41,5.07) |

© 2023 Nattino G et al. *JAMA Network Open.*

**Figure S.1.** Calibration belt of the logistic regression model estimating the probability of non-missing outcome.

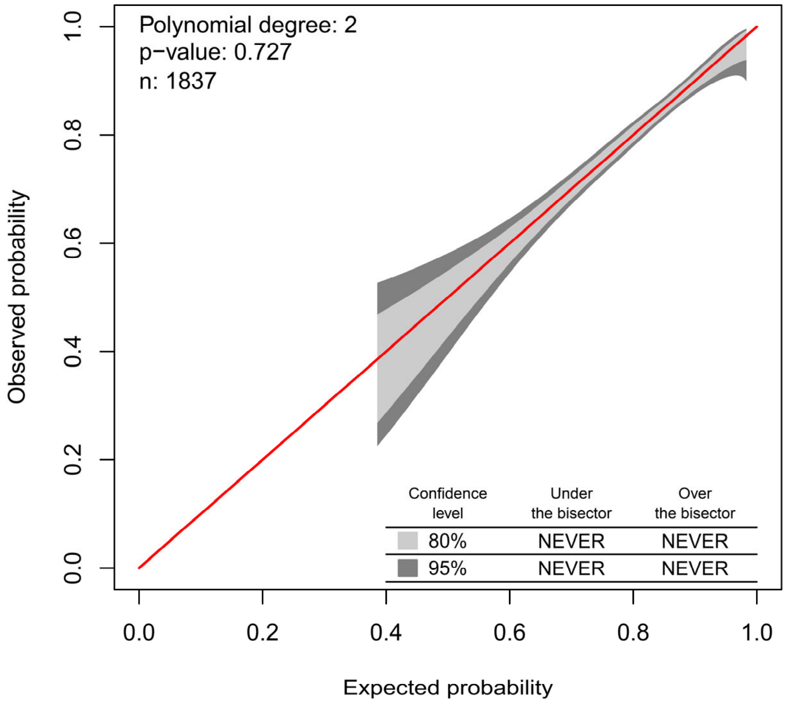


**Figure S.2.** Distribution of the estimated probabilities of non-missing outcome and corresponding weights, computed as the inverse of the probabilities.

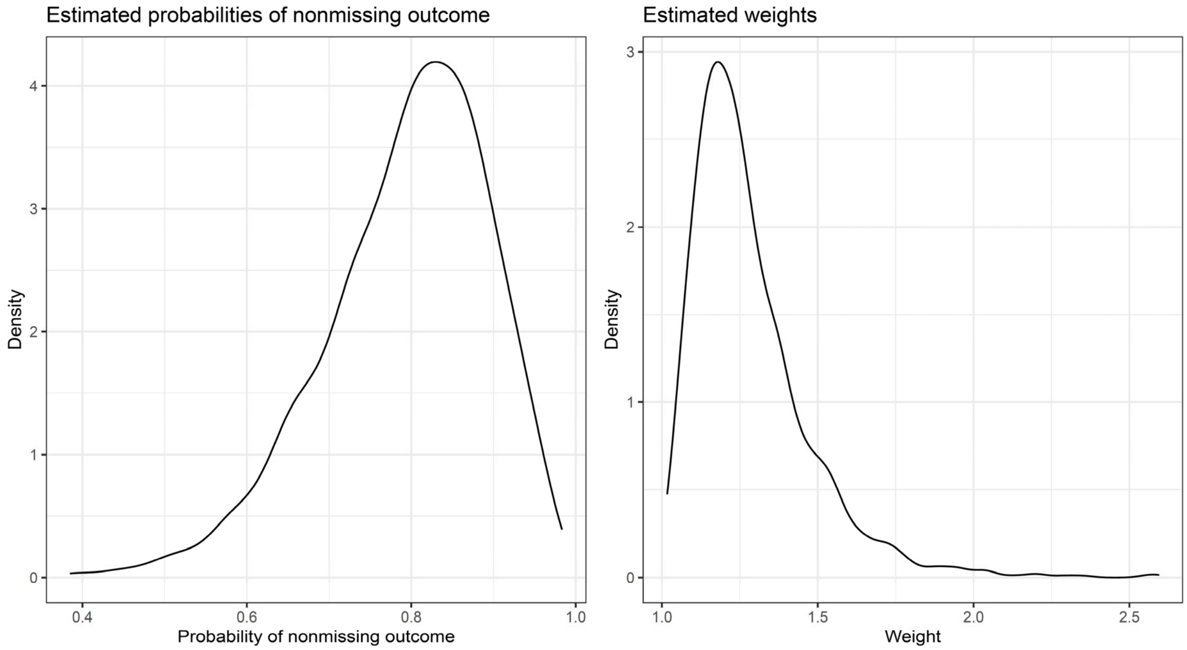


© 2023 Nattino G et al. *JAMA Network Open.*

**Table S.3.** Absolute weighted standardized mean differences (SMDs) comparing the treatment groups on all pre-treatment variables. The SMDs are weighted using the product of the matching and missing outcome weights.

| Variables | | Absolute weighted SMDs |
|---|---|---|
| Age | | 0.008 |
| Sex (Female) | | 0.085 |
| Comorbidities | | |
| | Antiplatelet therapy | 0.006 |
| | COPD | 0.003 |
| | Dementia | 0.013 |
| | Drug-induced coagulopathy | 0.020 |
| | Heart failure | 0.078 |
| | Liver disease | 0.072 |
| | Renal disease | 0.002 |
| Penetrating trauma | | 0.038 |
| Pre-treatment GCS | | 0.083 |
| Main lesion | | |
| | Cerebral contusion/laceration | 0.021 |
| | Extradural/epidural hematoma | 0.011 |
| | Traumatic subdural hematoma | 0.012 |
| | Intraparenchymal bleeding | 0.063 |
| | Diffuse injury without edema | 0.079 |
| | Diffuse injury with edema | 0.050 |
| | Subarachnoid hemorrhage | 0.011 |
| | Skull fracture | 0.031 |
| Injuries other than TBI | | |
| | Abdomen | 0.013 |
| | Chest | 0.054 |
| | Pelvis, bones, joints, and muscles | 0.051 |
| | Major vessels | 0.001 |
| | Spine | 0.059 |
| Pupils at ED arrival unilaterally dilated/non-reactive | | 0.005 |
| Hypotension | | 0.014 |
| Hypoxia | | 0.001 |
| Transfer from another ICU due to hospital expertise | | 0.033 |
| Surgery before ICU admission | | 0.013 |
| Neurosurgery within 2 days from injury | | 0.074 |
| Cardiovascular failure at ICU admission | | |
| | None | 0.025 |
| | Without shock | 0.061 |
| | With shock | 0.035 |
| Metabolic failure at ICU admission | | 0.033 |
| Renal failure at ICU admission | | 0.026 |
| Marshall CT classification | | |

© 2023 Nattino G et al. *JAMA Network Open.*

| Variables | | Absolute weighted SMDs |
| --- | --- | --- |
| | Diffuse Injury I | 0.020 |
| | Diffuse Injury II | 0.060 |
| | Diffuse Injury III | 0.059 |
| | Diffuse Injury IV | 0.044 |
| | Mass lesion (V or VI) | 0.009 |
| Midline shift >5mm | | 0.005 |
| Lesion volume >25ml | | 0.019 |
| Petechiae | | 0.061 |
| Cistern condition | | |
| | Normal | 0.095 |
| | Compressed or distorted | 0.093 |
| | Absent | 0.004 |

© 2023 Nattino G et al. *JAMA Network Open.*

**Table S.4.** Demographic and clinical characteristics at ICU admission of the patients after weighting for both matching and missing outcome weights.

| Variables | | No ICP monitoring | ICP monitoring | p-value |
|---|---|---|---|---|
| N | | 945 | 503 | |
| Weighted N | | 503 | 503 | |
| Age | | | | 0.90 |
| | Mean (SD) | 45.1 (18.2) | 45.0 (18.5) | |
| | Median (Q1-Q3) | 43.0 (29.0-58.0) | 44.0 (29.0-60.0) | |
| Sex (Female) – % | | 18.4% | 21.8% | 0.13 |
| Comorbidities – % | | | | |
| Any comorbidity | | 38.0% | 34.0% | 0.17 |
| Antiplatelet therapy | | 4.7% | 4.9% | 0.92 |
| COPD | | 2.2% | 2.2% | 0.96 |
| Dementia | | 0.3% | 0.4% | 0.97 |
| Drug-induced coagulopathy | | 2.4% | 2.7% | 0.67 |
| Heart failure | | 0.6% | 1.3% | 0.46 |
| Liver disease | | 3.4% | 2.2% | 0.22 |
| Renal disease | | 0.5% | 0.5% | 0.99 |
| Penetrating trauma – % | | 3.7% | 4.4% | 0.57 |
| Pre-treatment GCS – % | | | | 0.18 |
| | Mean (SD) | 5.1 (1.8) | 5.2 (1.8) | |
| | Median (Q1-Q3) | 5 (3-7) | 5 (3-7) | |
| Main lesion – % | | | | |
| | Cerebral contusion/laceration | 33.1% | 32.2% | 0.73 |
| | Extradural/epidural hematoma | 8.6% | 8.3% | 0.89 |
| | Traumatic subdural hematoma | 26.8% | 27.3% | 0.84 |
| | Intraparenchymal bleeding | 7.9% | 9.7% | 0.33 |
| | Diffuse injury without edema | 10.5% | 8.3% | 0.13 |
| | Diffuse injury with edema | 6.1% | 7.3% | 0.52 |
| | Subarachnoid hemorrhage | 6.3% | 6.5% | 0.84 |
| | Skull fracture | 0.6% | 0.4% | 0.94 |
| Injuries other than TBI – % | | | | |
| | Abdomen | 12.7% | 12.3% | 0.84 |
| | Chest | 39.9% | 42.5% | 0.40 |
| | Pelvis, bones, joints and muscles | 24.6% | 26.8% | 0.35 |
| | Major vessels | 3.6% | 3.6% | 0.98 |
| | Spine | 25.3% | 27.9% | 0.34 |
| | Other | 0.3% | 0.6% | 0.90 |
| Pupils at ED arrival – % | | | | 0.94 |
| | Bilaterally reactive/miotic | 67.4% | 67.6% | |
| | Unilaterally dilated/non-reactive | 32.6% | 32.4% | |
| Hypotension – % | | | | 0.93 |
| | Yes | 16.9% | 17.4% | |

© 2023 Nattino G et al. *JAMA Network Open.*

| Variables | No ICP monitoring | ICP monitoring | p-value |
|---|---|---|---|
| No | 78.6% | 77.8% | |
| Information not available | 4.5% | 4.8% | |
| Hypoxia – % | | | 0.76 |
| Yes | 33.7% | 33.7% | |
| No | 58.8% | 59.9% | |
| Information not available | 7.5% | 6.4% | |
| Transfer from other ICU for hospital expertise – % | 3.4% | 2.9% | 0.67 |
| Surgery before ICU admission – % | 59.5% | 60.1% | 0.83 |
| Neurosurgery within 2 days from injury – % | 41.6% | 45.2% | 0.28 |
| Cardiovascular failure on ICU admission – % | | | 0.62 |
| None | 41.3% | 40.0% | |
| Without Shock | 29.3% | 32.1% | |
| With Shock | 29.5% | 27.9% | |
| Metabolic failure on ICU admission – % | 25.4% | 24.0% | 0.60 |
| Renal failure on ICU admission – % | 6.6% | 7.3% | 0.64 |
| Worst CT scan of the first 48h in ICU – % | | | |
| Marshall scale | | | 0.73 |
| Diffuse Injury I | 4.2% | 3.8% | |
| Diffuse Injury II | 37.9% | 35.0% | |
| Diffuse Injury III | 13.8% | 15.9% | |
| Diffuse Injury IV | 2.6% | 3.4% | |
| Mass lesion (V or VI) | 41.4% | 41.8% | |
| Midline shift >5mm | 31.0% | 31.2% | 0.93 |
| Lesion volume >25ml | 32.9% | 33.8% | 0.78 |
| Petechiae | 48.7% | 51.8% | 0.36 |
| Cistern condition | | | 0.31 |
| Normal | 47.8% | 43.0% | |
| Compressed or distorted | 42.1% | 46.7% | |
| Absent | 10.2% | 10.3% | |

© 2023 Nattino G et al. *JAMA Network Open.*

**Table S.5.** Outcome of the patients after weighting for both matching and missing outcome weights.

| Variables | No ICP monitoring | ICP monitoring | p-value |
|---|---|---|---|
| N | 945 | 503 | |
| Weighted N | 503 | 503 | |
| ICU mortality – % | | | 0.79 |
| Alive | 76.1% | 76.7% | |
| Dead | 23.9% | 23.3% | |
| Hospital mortality – % | | | 0.50 |
| Alive | 70.1% | 71.8% | |
| Dead | 29.9% | 28.2% | |
| 6-month GOS-E – % | | | 0.003 |
| Dead (1) | 32.7% | 31.4% | |
| Vegetative State (2) | 5.8% | 6.0% | |
| Lower severe disability (3) | 17.7% | 25.1% | |
| Upper severe disability (4) | 7.3% | 8.5% | |
| Lower moderate disability (5) | 4.8% | 8.0% | |
| Upper moderate disability (6) | 11.2% | 8.3% | |
| Lower good recovery (7) | 7.7% | 5.9% | |
| Upper good recovery (8) | 12.8% | 6.8% | |

© 2023 Nattino G et al. *JAMA Network Open.*

**eAppendix 3. EXTENDED METHODS**

To address our research question, we applied a propensity-score-matched design. Using logistic regression, we calculated the propensity score, i.e., the probability of receiving ICP monitoring based on pre-treatment variables. As recommended (Stuart, *Matching Methods for Causal Inference: A Review and a Look Forward*, 2010), the variables included in the model were identified by a panel of clinicians (AG, LG, VC) based on a priori knowledge of the factors influencing both the decision to initiate ICP monitoring and the patient outcome. The panel deemed three of these factors essential to both the decision to start ICP monitoring and patient outcome: age group (<40, 40-64, 65-74, 75-79, ≥80), mass lesion (Marshall CT classification V or VI), and pre-hospital hypotension. Therefore, we matched patients on these same factors, i.e., we matched on the propensity score only patients with the same value for these three variables. To control for any bias that could have been introduced by an unbalanced distribution of the study countries, characterized by very different patient outcomes and TBI management policies, we also matched patients according to the country. We excluded patients from the countries where high-quality matching was not possible.

A 1:1 matched design was not feasible, as it was not possible to find one eligible control with a similar propensity score to each treated patient because of the distributions of the propensity score in the two groups. We opted for a full matching design, characterized by a series of matched sets with either one treated patient and multiple controls or one control and multiple treated patients (Rosenbaum, *A Characterization of Optimal Designs for Observational Studies*, 1991). The flexibility of this design facilitates the formation of well-balanced matched samples in conditions of poor overlap, where 1:1 matching algorithms are not appropriate. The matched sample was generated with the optimal full matching algorithm, implemented in the R package *optmatch* (Hansen and Klopfer, *Optimal Full Matching and Related Designs via Network Flows*, 2006). Following best practices, we excluded the control patients with an estimated propensity score lower than the minimum value of the treated group, being considered non-comparable to the treated patients in terms of pre-treatment covariates (Stuart and Rubin, *Best Practices in Quasi–Experimental Designs: Matching Methods for Causal Inference*, 2008).

Given the variable number of treated and control patients across matched sets, the post-matching analyses had to be weighted (Stuart and Green, *Using Full Matching to Estimate Causal Effects in Nonexperimental Studies: Examining the Relationship Between Adolescent Marijuana Use and Adult Outcomes*, 2008). As suggested, all treated subjects were assigned a weight of 1. In sets with one treated and k controls, controls were assigned a weight of 1/k, while in sets with k treated and one control, controls were assigned a weight of k. To avoid the formation of matched sets with a very large number of treated or control subjects, where controls would receive an overly large or small weight, we limited the maximum number of treated patients and controls to be included in each set to 5. All statistical analyses comparing the treatment groups of the matched sample were performed with weighted tests in weighted bivariate regression models, using the generated weights. Standard errors and p-values were computed using robust clustered standard errors, accounting for the possible correlation within the matched sets.

© 2023 Nattino G et al. *JAMA Network Open.*

**eAppendix 4. RESULTS OF THE ATTEMPT OF MATCHED ANALYSIS ON ALL COUNTRIES**

**Table S.6.** The table provides the results of the analysis in which we sought to generate a matched sample within the full cohort, including all the countries involved in the study. The table reports the absolute standardized mean differences (SMDs) before matching (left column) and the SMDs weighted by the matching weights. The presence of SMDs>10% for key confounders after weighting indicates that pre-treatment covariates are not adequately balanced. Such poor balance led to the selection of patients admitted to ICUs in Italy and Hungary, where control groups were larger.

| Variables | | SMDs before matching | Weighted SMDs after matching |
|---|---|---|---|
| Age | | 0.576 | 0.009 |
| Sex (Female) | | 0.132 | 0.072 |
| Comorbidities | | | |
| | Antiplatelet therapy | 0.202 | 0.002 |
| | COPD | 0.126 | 0.001 |
| | Dementia | 0.108 | 0.010 |
| | Drug-induced coagulopathy | 0.117 | 0.004 |
| | Heart failure | 0.063 | 0.041 |
| | Liver disease | 0.079 | 0.020 |
| | Renal disease | 0.117 | 0.025 |
| Penetrating trauma | | 0.070 | 0.014 |
| Pre-treatment GCS | | 0.068 | 0.042 |
| Main lesion | | | |
| | Cerebral contusion/laceration | 0.175 | 0.107 |
| | Extradural/epidural hematoma | 0.064 | 0.024 |
| | Traumatic subdural hematoma | 0.168 | 0.004 |
| | Intraparenchymal bleeding | 0.075 | 0.084 |
| | Diffuse injury without edema | 0.094 | 0.002 |
| | Diffuse injury with edema | 0.152 | 0.192 |
| | Subarachnoid hemorrhage | 0.155 | 0.028 |
| | Skull fracture | 0.037 | 0.081 |
| Injuries other than TBI | | | |
| | Abdomen | 0.015 | 0.012 |
| | Chest | 0.179 | 0.051 |
| | Pelvis, bones, joints and muscles | 0.019 | 0.060 |
| | Major vessels | 0.015 | 0.063 |
| | Spine | 0.111 | 0.045 |
| Pupils at ED arrival unilaterally dilated/non-reactive | | 0.075 | 0.017 |
| Hypotension | | 0.005 | 0.000 |
| Hypoxia | | 0.065 | 0.050 |
| Transfer from another ICU due to hospital expertise | | 0.053 | 0.075 |
| Surgery before ICU admission | | 0.303 | 0.089 |
| Neurosurgery within 2 days from injury | | 0.294 | 0.140 |
| Cardiovascular failure at ICU admission | | | |
| | None | 0.457 | 0.095 |

© 2023 Nattino G et al. *JAMA Network Open.*

| Variables | | SMDs before matching | Weighted SMDs after matching |
|---|---|---|---|
| | Without shock | 0.351 | 0.095 |
| | With shock | 0.151 | 0.003 |
| Metabolic failure at ICU admission | | 0.009 | 0.015 |
| Renal failure at ICU admission | | 0.190 | 0.009 |
| Marshall CT classification | | | |
| | Diffuse Injury I | 0.213 | 0.012 |
| | Diffuse Injury II | 0.014 | 0.147 |
| | Diffuse Injury III | 0.179 | 0.168 |
| | Diffuse Injury IV | 0.036 | 0.092 |
| | Mass lesion (V or VI) | 0.019 | 0.000 |
| Midline shift >5mm | | 0.097 | 0.010 |
| Lesion volume >25ml | | 0.006 | 0.019 |
| Petechiae | | 0.081 | 0.004 |
| Cistern condition | | | |
| | Normal | 0.004 | 0.180 |
| | Compressed or distorted | 0.116 | 0.193 |
| | Absent | 0.181 | 0.018 |

© 2023 Nattino G et al. *JAMA Network Open.*

**eAppendix 5. ICP MONITORING ACROSS ICUs**

**Figure S.2.** Number of eligible patients admitted to each Italian and Hungarian ICU, dividing monitored and nonmonitored patients.

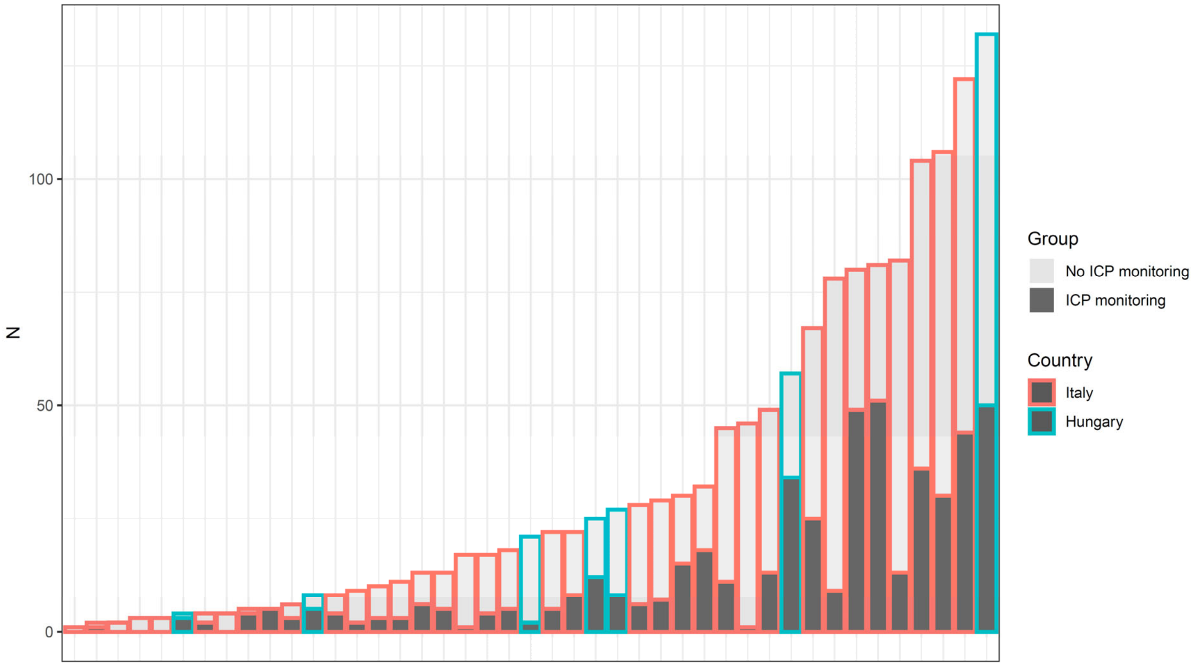


© 2023 Nattino G et al. *JAMA Network Open.*

**eAppendix 6. RESULTS OF THE MATCHED ANALYSIS ON ITALY AND HUNGARY**

**Table S.7.** Estimated odds ratios (ORs) and corresponding 95% confidence intervals of the logistic regression model estimating the propensity score.

| Variables | | OR (95% CI) |
|---|---|---|
| Age (10 year increase) | | 0.67 (0.62, 0.72) |
| Sex (Female vs. Male) | | 0.78 (0.58, 1.06) |
| Comorbidities | | |
| | Antiplatelet therapy (Yes vs. No) | 1.02 (0.61, 1.71) |
| | COPD (Yes vs. No) | 0.94 (0.46, 1.93) |
| | Dementia (Yes vs. No) | 0.22 (0.05, 1.02) |
| | Drug-induced coagulopathy (Yes vs. No) | 0.95 (0.49, 1.85) |
| | Heart failure (Yes vs. No) | 0.98 (0.37, 2.58) |
| | Liver disease (Yes vs. No) | 0.63 (0.29, 1.38) |
| | Renal disease (Yes vs. No) | 0.42 (0.12, 1.48) |
| Penetrating trauma (Yes vs. No) | | 1.02 (0.49, 2.12) |
| Pre-treatment GCS | | |
| | 3 | 0.92 (0.60, 1.40) |
| | 4-5 | 1.10 (0.71, 1.70) |
| | 6-7 | 0.94 (0.63, 1.40) |
| | 8 | 1 (ref.) |
| Main lesion | | |
| | Cerebral contusion/laceration | 3.85 (0.68, 21.94) |
| | Extradural/epidural hematoma | 2.07 (0.34, 12.60) |
| | Traumatic subdural hematoma | 1.93 (0.34, 11.07) |
| | Intraparenchymal bleeding | 3.30 (0.56, 19.36) |
| | Diffuse injury without edema | 2.02 (0.34, 11.89) |
| | Diffuse injury with edema | 4.62 (0.74, 29.02) |
| | Subarachnoid hemorrhage | 3.37 (0.59, 19.26) |
| | Skull fracture | 1 (ref.) |
| Injuries other than TBI | | |
| | Abdomen (Yes vs. No) | 0.67 (0.43, 1.04) |
| | Chest (Yes vs. No) | 1.35 (0.98, 1.85) |
| | Pelvis, bones, joints and muscles (Yes vs. No) | 0.91 (0.64, 1.28) |
| | Major vessels (Yes vs. No) | 1.16 (0.57, 2.35) |
| | Spine (Yes vs. No) | 1.32 (0.95, 1.85) |
| Pupils at ED arrival unilaterally dilated/non-reactive (Yes vs. No) | | 0.96 (0.71, 1.31) |
| Hypotension (Yes vs. No) | | 0.77 (0.53, 1.13) |
| Hypoxia (Yes vs. No) | | 0.99 (0.74, 1.32) |
| Transfer from another ICU due to hospital expertise (Yes vs. No) | | 1.76 (0.69, 4.46) |
| Surgery before ICU admission (Yes vs. No) | | 1.21 (0.80, 1.84) |
| Neurosurgery within 2 days from injury (Yes vs. No) | | 2.39 (1.47, 3.88) |
| Cardiovascular failure at ICU admission | | |
| | None | 1 (ref.) |
| | Without shock | 3.33 (2.42, 4.60) |

© 2023 Nattino G et al. *JAMA Network Open.*

| Variables | | OR (95% CI) |
|---|---|---|
| | With shock | 2.26 (1.57, 3.27) |
| Metabolic failure at ICU admission (Yes vs. No) | | 0.95 (0.69, 1.32) |
| Renal failure at ICU admission (Yes vs. No) | | 0.71 (0.45, 1.12) |
| Marshall CT classification | | |
| | Diffuse Injury I | 1 (ref.) |
| | Diffuse Injury II | 2.04 (0.85, 4.91) |
| | Diffuse Injury III | 2.61 (1.06, 6.41) |
| | Diffuse Injury IV | 3.67 (1.17, 11.52) |
| | Mass lesion (V or VI) | 2.21 (0.84, 5.81) |
| Midline shift >5mm (Yes vs. No) | | 0.41 (0.24, 0.69) |
| Lesion volume >25ml (Yes vs. No) | | 1.67 (0.96, 2.90) |
| Petechiae (Yes vs. No) | | 0.75 (0.56, 1.01) |
| Cistern condition | | |
| | Normal | 1 (ref.) |
| | Compressed or distorted | 1.85 (1.05, 3.27) |
| | Absent | 0.98 (0.51, 1.91) |

© 2023 Nattino G et al. *JAMA Network Open.*

**Table S.8.** Absolute weighted standardized mean differences (SMDs) comparing the treatment groups on all the pre-treatment variables. The SMDs are weighted using the weights defined by the full matching design.

| Variables | | SMDs before matching | Absolute weighted SMDs |
|---|---|---|---|
| Age | | 0.489 | 0.029 |
| Sex (Female) | | 0.071 | 0.090 |
| Comorbidities | | | |
| | Antiplatelet therapy | 0.120 | 0.004 |
| | COPD | 0.127 | 0.002 |
| | Dementia | 0.043 | 0.002 |
| | Drug-induced coagulopathy | 0.102 | 0.007 |
| | Heart failure | 0.003 | 0.079 |
| | Liver disease | 0.119 | 0.071 |
| | Renal disease | 0.095 | 0.003 |
| Penetrating trauma | | 0.117 | 0.042 |
| Pre-treatment GCS | | 0.062 | 0.099 |
| Main lesion | | | |
| | Cerebral contusion/laceration | 0.149 | 0.024 |
| | Extradural/epidural hematoma | 0.079 | 0.012 |
| | Traumatic subdural hematoma | 0.088 | 0.013 |
| | Intraparenchymal bleeding | 0.066 | 0.053 |
| | Diffuse injury without edema | 0.183 | 0.094 |
| | Diffuse injury with edema | 0.219 | 0.083 |
| | Subarachnoid hemorrhage | 0.181 | 0.024 |
| | Skull fracture | 0.073 | 0.013 |
| Injuries other than TBI | | | |
| | Abdomen | 0.016 | 0.016 |
| | Chest | 0.192 | 0.076 |
| | Pelvis, bones, joints and muscles | 0.003 | 0.033 |
| | Major vessels | 0.034 | 0.010 |
| | Spine | 0.115 | 0.055 |
| Pupils at ED arrival unilaterally dilated/non-reactive | | 0.032 | 0.027 |
| Hypotension | | 0.006 | 0.000 |
| Hypoxia | | 0.069 | 0.012 |
| Transfer from another ICU due to hospital expertise | | 0.060 | 0.005 |
| Surgery before ICU admission | | 0.277 | 0.004 |
| Neurosurgery within 2 days from injury | | 0.286 | 0.071 |
| Cardiovascular failure at ICU admission | | | |
| | None | 0.444 | 0.003 |
| | Without shock | 0.358 | 0.050 |
| | With shock | 0.141 | 0.048 |
| Metabolic failure at ICU admission | | 0.034 | 0.032 |
| Renal failure at ICU admission | | 0.122 | 0.029 |
| Marshall CT classification | | | |

© 2023 Nattino G et al. *JAMA Network Open.*

| Variables | | SMDs before matching | Absolute weighted SMDs |
| --- | --- | --- | --- |
| | Diffuse Injury I | 0.258 | 0.019 |
| | Diffuse Injury II | 0.173 | 0.068 |
| | Diffuse Injury III | 0.266 | 0.074 |
| | Diffuse Injury IV | 0.015 | 0.053 |
| | Mass lesion (V or VI) | 0.131 | 0.000 |
| Midline shift >5mm | | 0.016 | 0.019 |
| Lesion volume >25ml | | 0.110 | 0.004 |
| Petechiae | | 0.101 | 0.040 |
| Cistern condition | | | |
| | Normal | 0.196 | 0.084 |
| | Compressed or distorted | 0.295 | 0.081 |
| | Absent | 0.135 | 0.004 |

© 2023 Nattino G et al. *JAMA Network Open.*

**Table S.9.** Distribution of hospital and ICU characteristics of eligible patients.

| Variables | No ICP monitoring | | ICP monitoring | p-value[2] |
|---|---|---|---|---|
| | All | Weighted distribution[1] | | |
| N | 945 | 503 | 503 | |
| Vascular surgery – N (%) | 928 (98.2%) | 97.8% | 498 (99.0%) | 0.69 |
| Interventional neuroradiology – N (%) | 804 (90.4%) | 91.9% | 428 (89.5%) | 0.33 |
| Interventional vascular radiology – N (%) | 906 (95.9%) | 94.0% | 485 (96.4%) | 0.08 |
| Number of trauma patients/year | | | | 0.45 |
| Mean (SD) | 137.7 (66.8) | 134.2 (64.7) | 137.7 (67.5) | |
| Median (Q1, Q3) | 122.4 (91.2, 184.7) | 122.4 (90.8, 184.7) | 123.8 (92.4, 184.7) | |
| Number of TBI patients/year | | | | 0.14 |
| Mean (SD) | 76.5 (29.2) | 74.7 (27.4) | 77.5 (28.6) | |
| Median (Q1, Q3) | 73.5 (55.6, 93.3) | 73.5 (55.6, 93.3) | 82.2 (54.9, 93.3) | |

[1] Patients in the No ICP monitoring group are weighted, to make them comparable to patients in the ICP monitoring group with respect to pre-treatment covariates. Weights are defined by the matched design.
[2] P-value of the weighted tests comparing the no-ICP-monitoring and ICP monitoring groups.

© 2023 Nattino G et al. *JAMA Network Open.*

**eAppendix 7. COMORBIDITIES**

**Table S.10.** List of the comorbidities collected in the CREACTIVE study.

| Area | Comorbidity |
|---|---|
| Respiratory | Asthma |
| | Moderate COPD |
| | Severe COPD |
| | Restrictive lung disease |
| Cardiovascular | Arrhythmia |
| | Myocardiopathy |
| | Heart failure (NYHA class II-III or ACC stage C) |
| | Heart failure (NYHA class IV or ACC stage D) |
| | Myocardial infarction |
| | Hypertension |
| | Peripheral vascular disease |
| Neurologic | Dementia |
| | Hemiplegia or paraplegia or quadriplegia |
| | Cerebrovascular disease |
| | Neurodegenerative/Neuromuscular disease |
| Gastrointestinal and hepatic | Peptic ulcer disease |
| | Mild liver disease |
| | Moderate or severe liver disease |
| Renal | Moderate or severe renal disease |
| | End-stage renal disease |
| Endocrine | Diabetes Type I |
| | Diabetes Type II with insulin treatment |
| | Diabetes Type II without insulin treatment |
| Malignancy | Any tumour without metastasis |
| | Metastatic cancer |
| | Malignant hematological disease |
| Other | Autoimmune disease |
| | Immunosuppression |
| | Drug-induced coagulopathy |
| | Coagulation disorder |
| | Antiplatelet therapy |
| | AIDS |
| | Severe malnutrition |

© 2023 Nattino G et al. *JAMA Network Open.*

**eAppendix 8. INJURIES**

**Table S.11.** List of the injuries collected in the CREACTIVE study.

| Body region | Lesion |
|---|---|
| Spine | Cervical spinal cord injury with tetraplegia |
| | Cervical spinal cord injury with incomplete neurologic deficit |
| | Dorsal spinal cord injury with paraplegia |
| | Dorsal spinal cord injury with incomplete neurologic deficit |
| | Lumbar spinal cord injury with complete neurologic deficit |
| | Lumbar spinal cord injury with incomplete neurologic deficit |
| | Vertebral fracture without neurologic deficit |
| Chest | Major laceration of trachea/larynx |
| | Esophagus: rupture/perforation |
| | Traumatic emothorax and/or pneumothorax |
| | Traumatic massive hemothorax |
| | Tension pneumothorax |
| | Flail chest |
| | Severe lung contusion/laceration |
| | Cardiac trauma |
| | Diaphragmatic rupture |
| | Other injuries of the chest |
| Abdomen | Stomach: Rupture or perforation |
| | Bowel: Complete transection or perforation |
| | Pancreas: Laceration |
| | Liver: Moderate-severe laceration |
| | Liver: Massive laceration |
| | Spleen: Moderate-severe laceration |
| | Spleen: Massive rupture |
| | Kidney: Rupture/laceration |
| | Minor injuries of the abdomen |
| Pelvis, bones, joints and muscles | Long bone fracture |
| | Very severe or open fracture of the pelvis with unstable pelvis |
| | Multiple fracture of the pelvis |
| | Massive crush/amputation of one or more limbs |
| | Extremity compartment syndrome |
| Major vessels | Aorta: rupture/dissection |
| | Cava: rupture/transection |
| | Major thoracic vessels: transection |
| | Major abdominal vessels: transection |
| | Major vessels of the neck: dissection/transection |
| | Major vessels of the proximal limbs: transection |
| Other | Burns (> 30% of total body surface area) |
| | Inhalation injury |

© 2023 Nattino G et al. *JAMA Network Open.*
