# Supplementary material for: Comparative Effectiveness of Intracranial Pressure Monitoring on 6-Month Outcomes of Critically Ill Patients With Traumatic Brain Injury
Source: JAMA Netw Open. 2023 Sep 27;6(9):e2334214. doi: 10.1001/jamanetworkopen.2023.34214 (PMC10534270; doi:10.1001/jamanetworkopen.2023.34214)
Supplement: Supplement 2. — Nonauthor Collaborators [file jamanetwopen-e2334214-s002.pdf]

Supplemental Online Content: Nonauthor Collaborators for the CREACTIVE Consortium
*First name, last name, and suffix (if applicable) are required and will appear in PubMed.

## *Group Name(s): CREACTIVE consortium

| *First Name and Middle Initial(s) | *Last Name | *Suffix (eg, Jr, III) | Academic Degrees | Institution | Location (city, state/province, country) | Role or Contribution, eg, chair, principal investigator | Group (if more than 1 Group listed in the byline) and/or Subgroup (eg, Steering Committee) |
|---|---|---|---|---|---|---|---|
| Fulvio | Agostini | | | A.O.U. Città della Salute e della Scienza di Torino, Servizio Anestesia e Rianimazione 3 | Torino, Italy | | CREACTIVE consortium |
| Claudio | Ajmone-Cat | | | Azienda Ospedaliera San Camillo Forlanini, U.O.C. Shock e Trauma Centro di Rianimazione 1 | Roma, Italy | | CREACTIVE consortium |
| Giovanni | Bassi | | | Ospedale Civico Carrara, Servizio di Anestesia e Rianimazione | Carrara, Italy | | CREACTIVE consortium |
| Vasileios | Bekos | | | Athens Navy Hospital Intensive Care Unit | Athens, Greece | | CREACTIVE consortium |
| Marzia | Bellin | | | Ospedale dell'Angelo, Terapia Intensiva Generale | Mestre, Italy | | CREACTIVE consortium |
| Maria Grazia | Bocci | | | Policlinico Agostino Gemelli, Centro di Rianimazione | Roma, Italy | | CREACTIVE consortium |
| Valeria | Bonato | | | Civile - SS. Antonio e Biagio e C. Arrigo, Terapia Intensiva e Rianimazione | Alessandria, Italy | | CREACTIVE consortium |
| Alfeo | Bonato | | | Civil Hospital, Intensive Care Unit | Cittadella, Italy | | CREACTIVE consortium |
| Manuela | Bonizzoli | | | Azienda Ospedaliero Universitaria Careggi, Terapia Intensiva di Emergenza | Firenze, Italy | | CREACTIVE consortium |
| Paola | Bonucci | | | Azienda Ospedaliero - Universitaria Senese, Anestesia e Rianimazione DEA e dei Trapianti | Siena, Italy | | CREACTIVE consortium |
| Andrea | Bottazzi | | | Fondazione IRCCS Policlinico S.Matteo, Rianimazione Polivalente | Pavia, Italy | | CREACTIVE consortium |

Supplemental Online Content: Nonauthor Collaborators for the CREACTIVE Consortium
*First name, last name, and suffix (if applicable) are required and will appear in PubMed.

| ***First Name and Middle Initial(s)** | ***Last Name** | ***Suffix (eg, Jr, III)** | Academic Degrees | Institution | Location (city, state/province, country) | Role or Contribution, eg, chair, principal investigator | Group (if more than 1 Group listed in the byline) and/or Subgroup (eg, Steering Committee) |
|---|---|---|---|---|---|---|---|
| Italo | Calamai | | | Ospedale San Giuseppe | Empoli, Italy | | CREACTIVE consortium |
| Giuseppe | Calicchio | | | Azienda Ospedaliera Universitaria San Giovanni di Dio e Ruggi d'Aragona, Centro di Rianimazione | Salerno, Italy | | CREACTIVE consortium |
| Fabrizia | Carlin | | | Ospedale San Martino, Terapia Intensiva | Belluno, Italy | | CREACTIVE consortium |
| Sergio | Casagli | | | Azienda Ospedaliera Universitaria Pisana, S.D. Neuroanestesia/Rianimazione | Pisa, Italy | | CREACTIVE consortium |
| Carlo Alberto | Castioni | | | Ospedale San Giovanni Bosco, Terapia Intensiva | Torino, Italy | | CREACTIVE consortium |
| Rita | Ciceri | | | Ospedale A. Manzoni, Rianimazione Generale | Lecco, Italy | | CREACTIVE consortium |
| Francesco | Cocciolo | | | Ospedale Maurizio Bufalini, Anestesia e Rianimazione | Cesena, Italy | | CREACTIVE consortium |
| Ezio | Crestan | | | Azienda Ospedaliera Ospedale di Lecco, Neurorianimazione II SAR | Lecco, Italy | | CREACTIVE consortium |
| Gabor | Csato | | | Semmelweis University | Budapest, Hungary | | CREACTIVE consortium |
| Peter | Cseplo | | | Petz Aladár Megyei Oktató Kórház, Központi Aneszteziológiai és Intenzív Terápiás Osztály | Győr, Hungary | | CREACTIVE consortium |
| Francesco | Curto | | | ASST Grande Ospedale Metropolitano Niguarda, Neurorianimazione | Milano, Italy | | CREACTIVE consortium |
| Wojciech | Dąbrowski | | | University Hospital No 4, Anaesthesiology Intensive Therapy | Lublin, Poland | | CREACTIVE consortium |
| Anna | De Cristofaro | | | Azienda Ospedali Riuniti Marche Nord Presidio di Pesaro, Struttura Operativa Semplice di Rianimazione | Pesaro, Italy | | CREACTIVE consortium |

Supplemental Online Content: Nonauthor Collaborators for the CREACTIVE Consortium
*First name, last name, and suffix (if applicable) are required and will appear in PubMed.

| ***First Name and Middle Initial(s)** | ***Last Name** | ***Suffix (eg, Jr, III)** | Academic Degrees | Institution | Location (city, state/province, country) | Role or Contribution, eg, chair, principal investigator | Group (if more than 1 Group listed in the byline) and/or Subgroup (eg, Steering Committee) |
|---|---|---|---|---|---|---|---|
| Alessandra | De Luca | | | A.O. Universitaria Careggi, Area intensiva CTO | Firenze, Italy | | CREACTIVE consortium |
| Izabela | Duda | | | Uniwersyteckie Centrum Kliniczne im. prof. K. Gibińskiego Śląskiego Uniwersytetu Medycznego w Katowicach, Oddział Anestezjologii i Intensywnej Terapii | Katowice, Poland | | CREACTIVE consortium |
| Or | Duek | | | Ben Gurion University of the Negev, | Beer Sheva, Israel | | CREACTIVE consortium |
| Blanka Emoke | Bakó | | | Bács-Kiskun Megyei Kórház, Központi Aneszteziológia és Intenzív Terápiás Osztály | Kecskemét, Hungary | | CREACTIVE consortium |
| Nazzareno | Fagoni | | | Spedali Civili di Brescia, Neurorianimazione | Brescia, Italy | | CREACTIVE consortium |
| Paola | Fassini | | | Azienda Ospedale Civile di Legnano, Rianimazione | Legnano, Italy | | CREACTIVE consortium |
| Enrico | Ferri | | | Anesthesia Intensive Care and Prehospital Emergency, Maggiore Hospital | Bologna, Italy | | CREACTIVE consortium |
| Suada | Filekovic | | | University Medical Centre Ljubljana, Surgical ICU | Lubiana, Slovenia | | CREACTIVE consortium |
| Gilberto | Fiore | | | Santa Croce - Moncalieri - ASL TO 5, Rianimazione e Terapia Intensiva | Moncalieri, Italy | | CREACTIVE consortium |
| Emiliano | Gamberini | | | Ospedale Maurizio Bufalini, Unità Operativa di Anestesia e Rianimazione | Cesena, Italy | | CREACTIVE consortium |
| Diego | Gattari | | | Ospedale Civile, Rianimazione | Macerata, Italy | | CREACTIVE consortium |
| Massimo | Gianni | | | Ospedale Regionale Umberto Parini, Struttura Complessa di Rianimazione | Aosta, Italy | | CREACTIVE consortium |

*First name, last name, and suffix (if applicable) are required and will appear in PubMed.

| ***First Name and Middle Initial(s)** | ***Last Name** | ***Suffix (eg, Jr, III)** | Academic Degrees | Institution | Location (city, state/province, country) | Role or Contribution, eg, chair, principal investigator | Group (if more than 1 Group listed in the byline) and/or Subgroup (eg, Steering Committee) |
|---|---|---|---|---|---|---|---|
| Maria Giovanna | Dessena | | | Ospedale Giovanni Paolo II, Rianimazione | Olbia, Italy | | CREACTIVE consortium |
| Romano | Giuntini | | | Ospedale San Giuseppe, Rianimazione | Empoli, Italy | | CREACTIVE consortium |
| Stefania | Guido | | | AOU Maggiore della Carità, Anestesia e Rianimazione | Novara, Italy | | CREACTIVE consortium |
| Rita | Gyulai | | | Kenézy Kórház Gyula Kórház és Rendelőintézet, Központi Aneszteziológiai és Intenzív Terápiás Osztály | Debrecen, Hungary | | CREACTIVE consortium |
| Amir | Hadash | | | Rambam Medical Center, Pediatric Intensive Care Unit | Haifa, Israel | | CREACTIVE consortium |
| Renata | Hribar | | | Splošna Bolnišnica Novo Mesto, Enota Intenzivne Terapije | Novo Mesto, Slovenia | | CREACTIVE consortium |
| Stavroula | Ilia | | | University General Hospital of Heraklion Pagni, Pediatric Intensive Care Unit | Heraklion, Greece | | CREACTIVE consortium |
| Vesna Novak | Jankovic | | | Clinical Department of Anaesthesiology and Intensive Therapy, University Medical Centre Ljubljana | Ljubljana, Slovenia | | CREACTIVE consortium |
| Vlado | Jurekovic | | | General Hospital Jesenice | Jesenice, Slovenia | | CREACTIVE consortium |
| Mateja | Jereb | | | Clinical Department of Anaesthesiology and Intensive Therapy, University Medical Centre Ljubljana | Ljubljana, Slovenia | | CREACTIVE consortium |
| Maciej | Kapias | | | Szpital Śląski, Intensive Care Unit | Cieszyn, Poland | | CREACTIVE consortium |
| Dragica | Karadzic | | | Clinical Department of Anaesthesiology and Intensive Therapy, University Medical Centre Ljubljana | Ljubljana, Slovenia | | CREACTIVE consortium |

*First name, last name, and suffix (if applicable) are required and will appear in PubMed.

| ***First Name and Middle Initial(s)** | ***Last Name** | ***Suffix (eg, Jr, III)** | Academic Degrees | Institution | Location (city, state/province, country) | Role or Contribution, eg, chair, principal investigator | Group (if more than 1 Group listed in the byline) and/or Subgroup (eg, Steering Committee) |
|---|---|---|---|---|---|---|---|
| Darja | Kasnik | | | General Hospital Slovenj Gradec, Department for Anaesthesiology and Intensive Care Medicine | Slovenj Gradec, Slovenia | | CREACTIVE consortium |
| Volakakis | Vaggelis | | | Venizeleio - Pananeio General Hospital of Heraklion, Intensive Care Unit | Heraklion, Greece | | CREACTIVE consortium |
| Adrienn Kitti | Szaszi | | | Borsod-Abaúj-Zemplén Megyei Kórház és Egyetemi Oktató Kórház, Központi Aneszteziológiai és Intenzív Terápiás Osztály | Miskolc, Hungary | | CREACTIVE consortium |
| Janez | Kompan | | | General Hospital Slovenj Gradec, Department for Anaesthesiology and Intensive Care Medicine | Slovenj Gradec, Slovenia | | CREACTIVE consortium |
| Eraclis | Kyriakides | | | General Hospital | Nicosia, Cyprus | | CREACTIVE consortium |
| Silvia | Lagomarsino | | | Neurointensive Care Unit, Department of Anesthesia and Intensive Care Unit, AOU Careggi | Firenze, Italy | | CREACTIVE consortium |
| Sara | Lamborghini | | | Azienda Ospedaliero - Universitaria di Ferrara, Arcispedale S. Anna, Unità Operativa di Anestesia e Rianimazione Ospedaliera | Ferrara, Italy | | CREACTIVE consortium |
| Sergio | Livigni | | | Ospedale San Giovanni Bosco, Terapia Intensiva | Torino, Italy | | CREACTIVE consortium |
| Paolo | Malacarne | | | AOUP, Rianimazione / PS | Pisa, Italy | | CREACTIVE consortium |
| Maria | Martelli | | | AOUP, Rianimazione / PS | Pisa, Italy | | CREACTIVE consortium |
| Marina Alessandra | Martin | | | San Bortolo, Centro di Rianimazione | Vicenza, Italy | | CREACTIVE consortium |
| Costanza | Martino | | | Ospedale Maurizio Bufalini | Cesena, Italy | | CREACTIVE consortium |
| Andrea | Marudi | | | Nuovo Ospedale Civile Sant'Agostino Estense, Rianimazione Neurorianimazione | Modena, Italy | | CREACTIVE consortium |

Supplemental Online Content: Nonauthor Collaborators for the CREACTIVE Consortium
*First name, last name, and suffix (if applicable) are required and will appear in PubMed.

| ***First Name and Middle Initial(s)** | ***Last Name** | ***Suffix (eg, Jr, III)** | Academic Degrees | Institution | Location (city, state/province, country) | Role or Contribution, eg, chair, principal investigator | Group (if more than 1 Group listed in the byline) and/or Subgroup (eg, Steering Committee) |
|---|---|---|---|---|---|---|---|
| Martina | Melis | | | Ospedale Giovanni Paolo II, Rianimazione | Olbia, Italy | | CREACTIVE consortium |
| Francesca | Mengoli | | | Department of Integration, Palliative Care Network, Maggiore Hospital | Bologna, Italy | | CREACTIVE consortium |
| Tomislav | Mirkovic | | | Clinical Department of Anaesthesiology and Intensive Therapy, University Medical Centre Ljubljana | Ljubljana, Slovenia | | CREACTIVE consortium |
| Wiktoria | Mizak | | | Oddział Anestezjologii i Intensywnej Terapii, Szpital Powiatowy Lipsko | Lipsko, Poland | | CREACTIVE consortium |
| Marina | Munari | | | A.O. di Padova, Anestesia e Rianimazione NCH TI | Padova, Italy | | CREACTIVE consortium |
| Gabor | Nardai | | | Department of Anaesthesiology and Intensive Care, Péterfy Hospital and Trauma Centre | Budapest, Hungary | | CREACTIVE consortium |
| Ennio | Nascimben | | | Ospedale Ca' Foncello, Terapia Intensiva Neurochirurgica | Treviso, Italy | | CREACTIVE consortium |
| Giuseppe | Natalini | | | Fondazione Poliambulanza - Istituto Ospedaliero, Terapia Intensiva Polifunzionale | Brescia, Italy | | CREACTIVE consortium |
| Giancarlo | Negro | | | Francesco Ferrari, General Intensive Care Unit 1 | Casarano, Italy | | CREACTIVE consortium |
| Csaba | Nemes | | | Fejér Megyei Szent György Egyetemi Oktató Kórház, Központi Aneszteziológiai és Intenzív Betegellátó Osztály | Székesfehérvár, Hungary | | CREACTIVE consortium |
| Mara Olga | Bernasconi | | | S. Maria della Misericordia, Terapia Intensiva | Rovigo, Italy | | CREACTIVE consortium |

Supplemental Online Content: Nonauthor Collaborators for the CREACTIVE Consortium
*First name, last name, and suffix (if applicable) are required and will appear in PubMed.

| ***First Name and Middle Initial(s)** | ***Last Name** | ***Suffix (eg, Jr, III)** | Academic Degrees | Institution | Location (city, state/province, country) | Role or Contribution, eg, chair, principal investigator | Group (if more than 1 Group listed in the byline) and/or Subgroup (eg, Steering Committee) |
|---|---|---|---|---|---|---|---|
| Michele | Pagani | | | Fondazione IRCCS Policlinico S.Matteo, Rianimazione Polivalente | Pavia, Italy | | CREACTIVE consortium |
| Vieri | Parrini | | | Ospedale del Mugello, Anestesia e Rianimazione | Borgo San Lorenzo, Italy | | CREACTIVE consortium |
| Panagio | Partala | | | University Hospital Alexandroupolis, Intensive Care Unit | Alexandroupolis, Greece | | CREACTIVE consortium |
| Mauro | Pastorelli | | | Ospedale E. Agnelli, Rianimazione | Pinerolo, Italy | | CREACTIVE consortium |
| Isabella | Pellicioli | | | A.O. Papa Giovanni XXIII, Terapia Intensiva Pediatrica | Bergamo, Italy | | CREACTIVE consortium |
| Paolo | Perino Bert | | | Ospedale di Ciriè, Rianimazione e Terapia Intensiva | Torino, Italy | | CREACTIVE consortium |
| Nicola | Petrucci | | | Azienda Socio - Sanitaria Territoriale del Garda, Presidio di Desenzano, Rianimazione | Desenzano del Garda, Italy | | CREACTIVE consortium |
| Simone | Piva | | | Spedali Civili di Brescia, Rianimazione Polifunzionale 2 | Brescia, Italy | | CREACTIVE consortium |
| Daniele | Poole | | | Ospedale San Martino, Terapia Intensiva | Belluno, Italy | | CREACTIVE consortium |
| Laila | Portolani | | | Anesthesia and Intensive Care Unit, AUSL Romagna, Maurizio Bufalini Hospital | Cesena, Italy | | CREACTIVE consortium |
| Danilo | Radrizzani | | | Azienda Ospedale Civile di Legnano, Rianimazione | Legnano, Italy | | CREACTIVE consortium |
| Anna | Rekas | | | Wojewódzki Szpital Specjalistyczny, Intensive Care Unit | Lublin, Poland | | CREACTIVE consortium |
| Paweł | Robak | | | Szpital Miejski im.J.Strusia w Poznaniu, Oddział Anestezjologii i Intensywnej Terapii | Poznan, Poland | | CREACTIVE consortium |

Supplemental Online Content: Nonauthor Collaborators for the CREACTIVE Consortium
*First name, last name, and suffix (if applicable) are required and will appear in PubMed.

| ***First Name and Middle Initial(s)** | ***Last Name** | ***Suffix (eg, Jr, III)** | Academic Degrees | Institution | Location (city, state/province, country) | Role or Contribution, eg, chair, principal investigator | Group (if more than 1 Group listed in the byline) and/or Subgroup (eg, Steering Committee) |
|---|---|---|---|---|---|---|---|
| Antonio | Rosano | | | Fondazione Poliambulanza - Istituto Ospedaliero, Terapia Intensiva Polifunzionale | Brescia, Italy | | CREACTIVE consortium |
| Patrizia | Ruggeri | | | Istituti Ospitalieri di Cremona, Terapia Intensiva | Cremona, Italy | | CREACTIVE consortium |
| Marco | Sacchi | | | ASST Grande Ospedale Metropolitano Niguarda, Neurorianimazione | Milano, Italy | | CREACTIVE consortium |
| Ágnes | Sárkány | | | Fejér Megyei Szent György Egyetemi Oktató Kórház, Központi Aneszteziológiai és Intenzív Betegellátó Osztály | Székesfehérvár, Hungary | | CREACTIVE consortium |
| Mara | Skoti | | | Splosna Bolnisnica Izola, Enota Intenzivne Terapije | Izola, Slovenia | | CREACTIVE consortium |
| Alja | Skrt | | | Splosna Bolnisnica Izola, Enota Intenzivne Terapije | Izola, Slovenia | | CREACTIVE consortium |
| Ermanno | Spagarino | | | Nuovo Ospedale degli Infermi, Struttura Semplice di Rianimazione e Terapia Intensiva | Ponderano, Italy | | CREACTIVE consortium |
| Wiktor | Sulkowski | | | Szpital Powiatowy w Ostrowi Mazowieckiej, Anaesthesiology and Intensive Care Department | Ostrów Mazowiecka, Poland | | CREACTIVE consortium |
| Balázs | Szedlák | | | Borsod-Abaúj-Zemplén Megyei Kórház és Egyetemi Oktató Kórház, Központi Aneszteziológiai és Intenzív Terápiás Osztály | Miskolc, Hungary | | CREACTIVE consortium |
| Marina | Terzitta | | | G.B. Morgagni - L. Pierantoni, Rianimazione | Forlì, Italy | | CREACTIVE consortium |
| Rebecca | Tinturini | | | AOU Senese, Anestesia e Terapia Intensiva Neurochirurgia | Siena, Italy | | CREACTIVE consortium |

Supplemental Online Content: Nonauthor Collaborators for the CREACTIVE Consortium
*First name, last name, and suffix (if applicable) are required and will appear in PubMed.

| ***First Name and Middle Initial(s)** | ***Last Name** | ***Suffix (eg, Jr, III)** | Academic Degrees | Institution | Location (city, state/province, country) | Role or Contribution, eg, chair, principal investigator | Group (if more than 1 Group listed in the byline) and/or Subgroup (eg, Steering Committee) |
|---|---|---|---|---|---|---|---|
| Rossella | Tofani | | | Spedali Riuniti Livorno, Unità Operativa Anestesia e Rianimazione | Livorno, Italy | | CREACTIVE consortium |
| Paraskevi | Tselioti | | | Tzaneio General Hospital, General Intensive Care Unit | Pireus, Greece | | CREACTIVE consortium |
| Ada | Vecchiarelli | | | Santa Maria della Misericordia, Unità di Terapia Intensiva | Perugia, Italy | | CREACTIVE consortium |
| Elisabetta | Venturini | | | Civile - SS. Antonio e Biagio e C. Arrigo, Terapia Intensiva e Rianimazione | Alessandria, Italy | | CREACTIVE consortium |
| Salvatore | Visconti | | | Ospedale S. Maria di Loreto, Centro di Rianimazione "Marco Pica" | Napoli, Italy | | CREACTIVE consortium |
| Valeria | Zompanti | | | Ospedale Civile, Rianimazione | Macerata, Italy | | CREACTIVE consortium |
| Roberto | Zoppellari | | | Azienda Ospedaliero - Universitaria di Ferrara, Arcispedale S. Anna, Unità Operativa di Anestesia e Rianimazione Ospedaliera | Ferrara, Italy | | CREACTIVE consortium |
