# Supplementary material for: Comparative Effectiveness of Intracranial Pressure Monitoring on 6-Month Outcomes of Critically Ill Patients With Traumatic Brain Injury
Source: JAMA Netw Open. 2023 Sep 27;6(9):e2334214. doi: 10.1001/jamanetworkopen.2023.34214 (PMC10534270; doi:10.1001/jamanetworkopen.2023.34214)
Supplement: Supplement 3. — Data Sharing Statement [file jamanetwopen-e2334214-s003.pdf]

Data Sharing Statement

Nattino. Comparative Effectiveness of Intracranial Pressure Monitoring on 6-Month Outcomes of Critically Ill Patients With Traumatic Brain Injury. *JAMA Netw Open*. Published September 22, 2023. doi:10.1001/jamanetworkopen.2023.34214

Data
Data available: No
